# Optimized intrusion detection in IoT and fog computing using ensemble learning and advanced feature selection

**DOI:** 10.1371/journal.pone.0304082

**Published:** 2024-08-01

**Authors:** Mohammed Tawfik

**Affiliations:** Faculty of Computer and Information Technology, Sana’a University, Sana’a, Yemen; University of the West of Scotland, UNITED KINGDOM

## Abstract

The proliferation of Internet of Things (IoT) devices and fog computing architectures has introduced major security and cyber threats. Intrusion detection systems have become effective in monitoring network traffic and activities to identify anomalies that are indicative of attacks. However, constraints such as limited computing resources at fog nodes render conventional intrusion detection techniques impractical. This paper proposes a novel framework that integrates stacked autoencoders, CatBoost, and an optimised transformer-CNN-LSTM ensemble tailored for intrusion detection in fog and IoT networks. Autoencoders extract robust features from high-dimensional traffic data while reducing the dimensionality of the efficiency at fog nodes. CatBoost refines features through predictive selection. The ensemble model combines self-attention, convolutions, and recurrence for comprehensive traffic analysis in the cloud. Evaluations of the NSL-KDD, UNSW-NB15, and AWID benchmarks demonstrate an accuracy of over 99% in detecting threats across traditional, hybrid enterprises and wireless environments. Integrated edge preprocessing and cloud-based ensemble learning pipelines enable efficient and accurate anomaly detection. The results highlight the viability of securing real-world fog and the IoT infrastructure against continuously evolving cyber-attacks.

## 1 Introduction

The rapid expansion of the IoT has resulted in an epoch of billions of interconnected sensors, devices, and systems employed across a spectrum of industrial verticals, such as manufacturing, healthcare, transportation, energy, agriculture, smart cities, and homes [1, 2]. Forecasts predict that by 2025, over 30 billion IoT devices will be operational worldwide, facilitating a broad range of applications from remote asset monitoring to autonomous systems, thus birthing colossal hyperconnected ecosystems [3–5]. This massive network of smart interconnected IoT devices and sensors amplifies automation, efficiency, and real-time data collection and analytics at the granular level [6]. However, these IoT networks’ broad scale and distribution also significantly broaden the potential attack surface for a myriad of cybersecurity threats from external attackers and malicious insiders [7].

IoT systems are inherently challenged by device constraints in terms of limited computing power, memory, storage capacity, and battery life [8]. These limitations critically hinder the feasibility of deploying conventional complex security algorithms and protocols directly on the IoT devices. Concurrently, traditional cloud-centric security architectures encounter multiple limitations when tailored to secure diverse IoT deployments that encompass thousands of endpoints across various geographic locations [9]. Issues, such as latency, mobility support, and geographic distribution render cloud-based security mechanisms impractical for many IoT applications [10]. This scenario necessitates the inception of innovative decentralised security paradigms to address the unique needs and constraints of IoT ecosystems [11].

Fog computing has emerged as an encouraging new framework that strengthens IoT networks by implementing a distributed and decentralized architecture [12]. In fog computing, a hierarchical intermediate layer of fog nodes connects cloud data centres and IoT end devices stationed at the network edge [13]. These fog nodes comprise an array of devices, including gateways, routers, switches, and microdata centres, all endowed with computing, storage, networking, and security capabilities [14]. The essence of fog computing lies in dispersing intelligence closer to the data source, thereby serving latency-sensitive IoT applications more efficiently [15]. Fog nodes offer decentralized computing, storage, and networking resources, enabling various security functionalities such as authentication, access control, data encryption, anomaly detection, and intrusion prevention to be deployed in close proximity to IoT devices [16]. This edge-centric model strives to overcome the challenges of cloud dependence for budding IoT use cases in domains such as smart grids, intelligent transportation, healthcare systems, and industrial control networks [17].

However, fortifying fog-computing architectures against evolving cyber threats presents unique challenges [18]. Fog nodes are generally resource-constrained compared to cloud servers in terms of computing capacity, memory, storage, and battery power [19]. Deploying computationally intensive security algorithms to fog nodes can hinder their performance and thwart their mainstream adoption [20]. The distributed architecture of fog computing requires collaborative threat detection and response across various heterogeneous fog nodes [21]. Reliability and fault tolerance are also critical considerations for ensuring resilient operation during intermittent connectivity between fog nodes at various locations [22]. Security issues in the real world, including DDoS attacks and other malware infections, have exposed the practical vulnerabilities of critical IoT infrastructures reliant on fog computing [23]. This highlights the crucial need for robust intrusion detection capabilities tailored to fog-computing environments.

Intrusion detection systems (IDS) fulfill the essential role of identifying unauthorized access through the monitoring of intrusive behaviors. For instance, host-based intrusion detection systems (HIDS) focus on individual host activities, whereas network-based intrusion detection systems (NIDS) are typically positioned at network junctions such as gateways or routers to inspect traffic for intrusion signs. Because fog computing architectures are blossomed, intrusion detection techniques have become indispensable for providing comprehensive monitoring, analysis, and response through continuous tracking of events and activities across fog networks [24].

IDS identifies unauthorized access by monitoring intrusive behaviors [25]. There are different categories of IDS technology. Host-based IDS (HIDS) focuses specifically on host-level activities and events rather than on network traffic. Detects attacks targeting vulnerabilities in operating systems and applications. Network-based IDS (NIDS) are typically placed at network junctions such as gateways and routers. They analyze network packets and traffic patterns to identify attacks. As fog-computing architectures have proliferated globally, IDS solutions have become vital for securing infrastructure against continuously evolving threats. The unique distributed nature of fog networks creates additional challenges for attack monitoring and analysis [26]. Effective IDS techniques for fog computing must provide the following:

- Continuous tracking of malicious events and activities
- Centralized monitoring across heterogeneous fog nodes
- Real-time attack detection and response
- Resilient threat analytics despite intermittent connectivity

Robust IDS tailored to handle such constraints remains an open research challenge. Exploring machine learning and evolutionary computation methods shows promise for developing more adaptive IDS for resource-limited fog environments [27]. IDS solutions bolster fog nodes against attacks by identifying anomalous patterns and devising appropriate response strategies based on observed attacker behaviors. However, most existing IDS techniques and tools have been crafted for cloud or enterprise environments using signature-based detection, and adapting these conventional approaches to function in resource-constrained fog nodes effectively presents significant challenges. Developing IDS techniques, particularly those optimized for the unique characteristics of fog computing, remains an open research challenge. Customized IDS solutions employing machine learning, deep learning, and evolutionary computation have yielded promising results. However, critical issues remain, including optimal feature engineering, model complexity reduction, real-time detection of fog hardware, and collaborative learning across the federated fog nodes. Further exploration is imperative to address these limitations using an integrated intrusion detection framework encompassing data preprocessing, feature extraction, classification, and cross-layer optimization techniques tailored for resource-constrained fog environments.

The objective of this research is to develop an optimized and efficient intrusion detection system that addresses the unique security challenges of fog computing and IoT networks. The proposed framework aims to leverage the distributed nature of fog architectures while considering the resource constraints of fog nodes and the heterogeneity of IoT devices. By integrating advanced feature engineering techniques, deep learning architectures, and bio-inspired optimization algorithms, this study seeks to push the frontiers of intelligent, adaptive, and collaborative intrusion detection tailored for the fog-IoT ecosystem. The ultimate goal is to contribute towards the realization of secure, resilient, and self-healing IoT infrastructures capable of autonomously detecting and mitigating evolving cyber threats in real time.

This study proposes a unique intrusion detection framework with the following specific contributions:

1. An integrated pipeline combining stacked autoencoders for efficient fog-based feature extraction, CatBoost for predictive edge feature selection, and an optimized transformer-CNN-LSTM cloud-hosted ensemble classifier for anomaly detection.
2. Customization of the Transformer, CNN, and LSTM architectures tailored for network intrusion detection through synergistic integration rather than off-the-shelf deployment.
3. An adaptive grey wolf optimization strategy was employed for the fine-tuning of the ensemble model’s hyperparameters, with the aim of enhancing the classification performance across a variety of network traffic datasets.
4. Evaluations of benchmarks, such as NSL-KDD, demonstrate state-of-the-art accuracy, F1 scores, and ROC-AUC for identifying threats in traditional, enterprise, and wireless environments.

The proposed innovations in deep ensemble architecture design, biologically inspired optimization, and edge-cloud synergy help push the frontier towards securing emerging fog computing and IoT infrastructures against continuously evolving cyberattacks. By fostering more robust detection capabilities, this study contributes to the realization of intelligent self-healing IoT ecosystems.

The rest of the paper is organized as follows: Section 2 presents related work, Section 3 describes the proposed methodology, Section 4 details the experimental setup, Section 5 discusses the results and analysis, and Section 6 concludes the paper with future research directions.

## 2 Related work

Intrusion detection systems (IDS) are essential cybersecurity technologies that scrutinize network traffic and system activities to identify malicious behaviors and cyber threats [28]. IDS have become increasingly crucial for safeguarding fog computing and IoT infrastructures against continuously evolving and sophisticated cyberattacks. Fog computing represents an architecture that broadens the abilities of cloud computing to the peripheries of networks, thereby providing low-latency computing services to endpoint IoT devices [29]. However, resource constraints and the distributed nature of fog and IoT environments make intrusion detection considerably more challenging than in traditional networks.

Over the past decade, a significant amount of research has been dedicated to devising optimized ML and DL strategies explicitly for IDS on fog nodes with limited resources. Conventional shallow ML models, such as SVM, DT, RF, Naive Bayes classifiers, and ensemble methods, have undergone thorough evaluation on benchmark IDS datasets, such as UNSW-NB15 and NSL-KDD [1, 28, 29]. For instance, Al-Yaseen et al. [1] achieved a detection accuracy of over 90% on the NSL-KDD dataset using hierarchical multilevel models based on algorithms such as C5.0, random forest, and SVM. However, these conventional shallow learning architectures often face limitations in identifying the more complex and emerging attack patterns seen in modern cyber threats [1, 30–33].

Advanced deep learning models such as CNN, LSTM networks, various autoencoder architectures, and hybrid combinations of these have shown tremendous promise for significantly advancing the capabilities of IDS [28, 32, 34–37]. In particular, LSTM networks leverage their innate sequence-learning strengths to model the temporal relationships found in network traffic effectively. LSTM-based approaches have achieved approximately 99% accuracy on the UNSW-NB15 and NSL-KDD benchmarks, outperforming conventional techniques such as SVM and RF classifiers [29]. CNN can extract high-level discriminative features from raw network traffic data through the application of convolutional filters, which is highly beneficial for multiclass intrusion detection [37–39].

Recently, hybrid models combining diverse complementary neural architectures have become popular. For instance, Surya et al. [40] developed a hybrid LSTM-DL model tailored for fog-computing intrusion detection, which attained an accuracy of 99.2% on the NSL-KDD dataset. Integrating different types of deep networks allows for learning enriched representations of network data compared with single-architecture models [37, 41, 42].

Ensemble techniques that aggregate the outputs from diverse machine learning models, such as SVM, DT, and RF classifiers, have also been extensively explored to improve the generalization capabilities compared with individual models. For example, Khammassi and Krichen [29] presented an ensemble approach that achieved 99.8% accuracy on the KDDCUP99 benchmark through synergistic integration of the SVM, RF, and DT models. Such classifier ensembles aim to overcome the limitations of the individual models.

Swarm intelligence-based optimization algorithms and other evolutionary computing methods enable the optimization of key intrusion detection system parameters in areas such as feature selection, model hyperparameters, and ensemble configurations [43]. Shubhra et al. utilized an artificial grasshopper optimization technique to significantly boost the accuracy of an SVM classifier to 99.6% on the KDDCUP99 dataset [44]. These metaheuristic algorithms allow joint optimization of multiple facets of the IDS design for maximum performance.

Dimensionality reduction techniques and feature selection are also critical preprocessing steps for deriving optimal and minimal subsets of relevant input features, which facilitates more efficient learning [45–48]. Stacked autoencoders (SAEs) are widely used in unsupervised feature learning and dimensionality reduction to improve intrusion detection, and Muhammad et al. [49] proposed the use of SAE with two latent layers, succeeded by a supervised deep neural network classifier. When evaluated on the KDDCup99, NSL-KDD, and AWID intrusion detection datasets, their model achieves 94.2-99.9% accuracy for multiclass classification across different attack types. This demonstrates that SAEs can effectively learn representative features from network traffic data, and when combined with deep neural network classifiers, high-performance network intrusion detection systems can be built.

More recent research has started to focus specifically on tailoring deep learning advances to IoT environments for cybersecurity, as IoT devices proliferate globally [50–52]. Soe et al. [50] applied artificial neural networks for DDoS attack recognition in IoT environments. Shafiq et al. [51] utilized an SVM, decision trees, and RF classifiers to detect IoT botnets, achieving an attack detection accuracy of up to 97%. Autoencoders have shown promise in feature learning and dimensionality reduction for enhancing anomaly detection in network IDS. Khan and Mailewa [53] proposed a hybrid deep autoencoder (DAE) and SVM model. When evaluated on the NSL-KDD intrusion detection dataset, their DAE-SVM model attains a micro-average F1-score of 0.72 for multi-class attack detection, compared to 0.63 for a PCA-SVM baseline. The autoencoder provides an effective nonlinear feature fusion before the SVM classifier. Overall, the DAE-SVM model outperformed the PCA-SVM and standalone SVM classifiers in terms of accuracy, F1-score, precision, recall, and computational overhead for detecting anomalies across different network attack types. Lilhore et al. [54] proposed a novel security framework leveraging deep learning for intrusion detection in IoT and 5G networks. Their hybrid model combines MobileNetV3-SVM architecture and Transfer Learning to efficiently analyze network activity and identify breaches in real-time. The MobileNetV3 framework is used for feature extraction, optimized for IoT devices, while SVM is applied for classification. The model’s performance is evaluated using various metrics, demonstrating its superiority over existing models like VGG-16, VGG-19, Efficient-Net, and Inception-Net across multiple IDS datasets. On the CICIDS-2017 dataset, the proposed method achieved remarkable accuracy rates for different attack classes, such as 98.98% for ‘WebAttack’, 99.01% for ‘DOS’, and 98.74% for ‘DDOS’. This research contributes to enhancing the adaptability of security measures to evolving threat vectors and traffic characteristics in dynamic IoT and 5G systems.

For fog computing architectures, Abeshu and Chilamkurti [55] designed a distributed deep-learning framework using stacked autoencoders for intrusion detection tailored to fog networks, achieving a 99% detection accuracy on a custom fog computing dataset [20]. Diro and Chilamkurti [21] presented a long short-term memory network-based approach for fog attack detection evaluated using the ISCX dataset. Keshk et al. [56] introduced a privacy-preserving LSTM model for fog nodes that incorporated blockchain technology coupled with variational autoencoders to enhance security.

The integration of diverse deep neural architectures, ensemble machine learning techniques, nature-inspired optimization algorithms, and unsupervised feature learning methods has immense potential to meaningfully improve the capabilities of IDS for resource-constrained fog computing and IoT environments [55–57]. A unified synergistic framework that comprehensively combines these promising approaches could provide next-generation IDS with substantially improved accuracy, efficiency, adaptability, and generalization abilities. Our proposed research aims to develop an optimized ensemble IDS that integrates convolutional and recurrent networks, autoencoders for feature extraction, and evolutionary multi-objective optimization to achieve state-of-the-art performance in cybersecurity for fog computing and the IoT infrastructure.

## 3 Proposed intrusion detection system

The proposed methodology is designed to address the unique challenges of intrusion detection in fog computing and IoT networks. The integration of stacked autoencoders (SAEs), CatBoost, and an optimized transformer-CNN-LSTM ensemble is tailored to handle the resource constraints of fog nodes while leveraging the computational power of the cloud for comprehensive threat analysis.

SAEs are chosen for their ability to extract robust features from high-dimensional network traffic data while reducing dimensionality, making them well-suited for the limited resources of fog nodes [51]. The unsupervised nature of SAEs allows for learning representative features without the need for labelled data, which is often scarce in real-world scenarios. CatBoost, a gradient boosting algorithm, is selected for its efficiency in handling categorical features and its ability to rank feature importance [61]. This enables the identification of the most predictive features, further optimizing the computational resources at the edge.

The transformer-CNN-LSTM ensemble is designed to capture both global and local patterns in network traffic sequences. Transformers excel at modeling long-range dependencies through self-attention [62], while CNNs are effective in extracting local spatial features [37]. LSTMs provide the ability to learn temporal relationships [41]. By combining these complementary architectures, the ensemble model can comprehensively analyze network activity and identify complex attack patterns.

The use of Adaptive Grey Wolf Optimization (AGWO) for hyperparameter tuning ensures that the ensemble model is optimized for maximum performance [63]. AGWO efficiently explores the hyperparameter space, adapting to the intrusion detection task’s specific requirements and the characteristics of the fog-IoT environment.

By integrating these techniques in a fog-cloud collaborative framework, the proposed methodology addresses the key issues of resource efficiency, real-time detection, and adaptability to evolving threats. The distribution of tasks between the edge and the cloud allows for a balance between local real-time analysis and global threat intelligence, providing a comprehensive solution for intrusion detection in fog computing and IoT networks.

This study proposes a unique intrusion detection framework with the following specific contributions:

1. An integrated pipeline combining stacked autoencoders for efficient fog-based feature extraction, CatBoost for predictive edge feature selection, and an optimized transformer-CNN-LSTM cloud-hosted ensemble classifier for anomaly detection.
2. Customization of the Transformer, CNN, and LSTM architectures tailored for network intrusion detection through synergistic integration rather than off-the-shelf deployment.
3. An adaptive grey wolf optimization strategy was employed for the fine-tuning of the ensemble model’s hyperparameters, with the aim of enhancing the classification performance across a variety of network traffic datasets.
4. Evaluations of benchmarks, such as NSL-KDD, demonstrate state-of-the-art accuracy, F1 scores, and ROC-AUC for identifying threats in traditional, enterprise, and wireless environments.

The proposed innovations in deep ensemble architecture design, biologically inspired optimization, and edge-cloud synergy help push the frontier toward securing emerging fog computing and IoT infrastructures against continuously evolving cyberattacks. By fostering more robust detection capabilities, this study contributes to the realization of intelligent self-healing IoT ecosystems.

**Fig 1:**
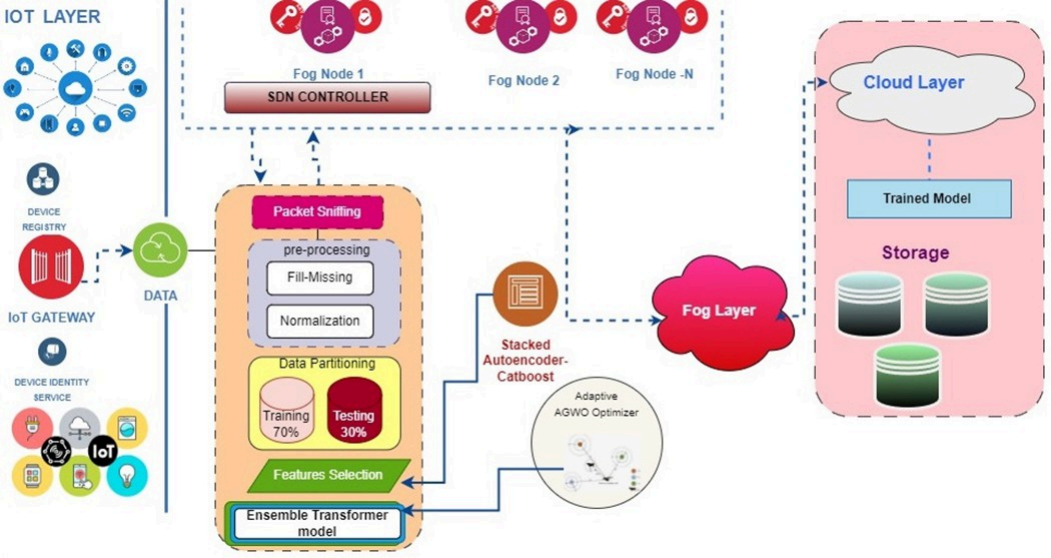
System architecture diagram illustrating the proposed intrusion detection framework. It depicts fog nodes at the network edge that collect and preprocess IoT sensor data using stacked autoencoders. The compressed analytic features from the fog are relayed to the cloud for detection using an optimized deep-learning ensemble model. The framework combines localized real-time anomaly detection in fog with holistic intrusion analysis in the cloud.

By synergizing edge preprocessing with cloud-based deep learning, our system aims to deliver an intelligent distributed IDS tailored to IoT environments, harnessing the advantages of both fog and cloud computing. The system being proposed is depicted in Fig 1.

### 3.1 Preprocessing

Prior to building and training deep neural network models for intrusion detection, raw network traffic datasets undergo a multistep preprocessing pipeline to transform the data into formats suitable for effective learning [62]. The key preprocessing steps are as follows:

#### 3.1.1 Data cleansing

First, the datasets were checked for missing values and incomplete data. Any corrupted, duplicate, or erroneous data points were removed or fixed to ensure high-quality data for the model training.

#### 3.1.2 Label encoding

Categorical features in the dataset, such as protocol types or service names, were label encoded to numeric representations. This involved mapping each unique text value onto a numerical label. Encoding transforms categories into a format that is understandable to deep-learning models.

#### 3.1.3 Standard scaling

Numeric features such as time duration or byte count were standardized using a standard scalar. This process transforms the features into a standard distribution with a mean of 0 and a variance of 1 using the following formula:

$$\begin{array}{c} z=\frac{x-\mu }{\sigma } \end{array}$$ (1)

where *z* is the scaled value, *x* is the initial value, *μ* is the mean, and *σ* is the standard deviation. Standardization places all numeric features into a common range and scale that is suitable for effective model training.

### 3.2 Feature extraction and selection for model optimization

Feature selection is the process of detecting and choosing a smaller number of important features from a larger set, depending on their capacity to maximize the distinction between class labels. In the context of machine learning, feature selection involves selecting a subset of important features based on specific criteria. Real-world datasets often contain redundant, irrelevant, and noisy attributes [58], making feature selection crucial for improving efficiency and reducing dimensionality.

Feature engineering is critical in constructing machine learning models that achieve high performance, particularly when working with complex, high-dimensional data. This section presents a robust feature engineering workflow tailored to optimize an IoT intrusion detection model. The pipeline leverages Stacked Autoencoders (SAEs) for unsupervised nonlinear feature reduction and CatBoost for supervised predictive feature selection.

#### 3.2.1 Stacked autoencoder for dimensionality reduction

The network traffic dataset contains numerous features with redundancies and irrelevant attributes that can negatively impact model performance. SAEs are a type of DNN composed of multiple layers of autoencoders for learning useful data representations in an unsupervised manner [59]. Autoencoders are trained to encode the input data into a compressed depiction and then decode that representation back into the previous input space. As described by Bengio [60], stacked autoencoders involve stacking shallow autoencoders using the output code from one autoencoder as the input for the next autoencoder. Specifically, the encoder portion of the first autoencoder compresses the input data. The result is then fed to the second autoencoder that learns another compressed representation. This process was repeated for the additional layers. Finally, decoder networks reconstruct the original input from the deepest representation.

A key motivation for stacking autoencoders is to learn a categorized feature representation, in which simple features are extracted in the early layers, whereas deeper layers focus on more complex, abstract concepts. As data are progressively encoded and decoded, the model is forced to prioritize aspects that must be preserved for accurate reconstruction. This tendency to learn the intrinsic manifold of the data makes stacked autoencoders very useful for unsupervised feature learning.

The optimization of stacked autoencoders involves minimizing the reconstruction error between the initial and final outputs. Common loss functions include mean squared error and mean absolute error. Appropriate regularization techniques help prevent overfitting. Careful hyperparameter tuning of depth, width, activation functions, and training procedures is necessary for good performance. To obtain the most salient information, a deep Stacked Autoencoder (SAE) was used for nonlinear dimensionality reduction.

SAEs are neural networks comprising multiple layers of encoders and decoders. Encoders transform the input into smaller hidden representations through nonlinear activation. Decoders attempt to reconstruct the original input from the hidden code. By training the SAE to minimize the reconstruction error, the smallest bottleneck layer learns to get the most important properties of the records as low-dimensional robust features: hierarchical nonlinear feature learning, capturing multi-level data patterns, good generalization to new data, compression of high-dimensional inputs, and unsupervised feature extraction.

These characteristics make SAEs well-suited for learning informative nonlinear feature representations from complex network traffic data in an unsupervised manner. The architecture comprises multiple encoding layers to transform the input into successively smaller codes, a bottleneck layer with the smallest dimensions, and symmetric decoding layers to reconstruct the original input. The training objective was to minimize the reconstruction loss between the input and output.

$$\begin{array}{c} L\left(x,x^{\prime }\right)={\left\|x-x^{\prime }\right\|}^{2} \end{array}$$ (2)

The SAE was implemented in Keras using dense layers for the encoders and decoders. The dimensions of the hidden layers were progressively reduced from the input size to the bottleneck size. ReLU activation is used for nonlinear encoding. The model underwent training with the objective of reducing the mean squared error reconstruction loss using the Adam optimizer for 30 epochs.

A random search sampled 50 different hyperparameter configurations from predefined ranges, and evaluated each configuration based on the validation loss. The optimal settings were used to retrain the final SAE model for feature extraction.

*Encoding phase*. For a three-layer encoder, the transformations can be represented as follows. In the encoding phase, the input data *X* is transformed into a more compact representation through a series of layers. Each layer *h*_*i*_ is computed as a function *f*_*i*_ of the previous layer’s output (or the input for the first layer) and the layer’s parameters *w*_*i*_ and *b*_*i*_:

$$\begin{array}{c} h_{1}=f_{1}\left(W_{1}x+b_{1}\right) \end{array}$$ (3)

This represents the first encoding layer, where the input *X* is linearly transformed by weights *W*_1_ and biases *b*_1_, followed by a nonlinear activation function *f*_1_.

$$\begin{array}{c} h_{2}=f_{2}\left(W_{2}h_{1}+b_{2}\right) \end{array}$$ (4)

The second layer takes the output of the first layer *h*_1_, applies another set of linear transformation with its own parameters *W*_2_ and *b*_2_, then a second activation function.

$$\begin{array}{c} h_{3}=f_{3}\left(W_{3}h_{2}+b_{3}\right) \end{array}$$ (5)

*Decoding phase*. The decoding phase attempts to reconstruct the original input *X* from the encoded representation *h*_3_:

$$\begin{array}{c} h_{4}=f_{4}\left(W_{4}h_{3}+b_{4}\right) \end{array}$$ (6)

$$\begin{array}{c} h_{5}=f_{5}\left(W_{5}h_{4}+b_{5}\right) \end{array}$$ (7)

$$\begin{array}{c} x^{\prime }=f_{6}\left(W_{6}h_{5}+b_{6}\right) \end{array}$$ (8)

*Reconstruction loss*. The SAE undergoes training with the aim of minimizing the reconstruction error between the input *x*′, which is often computed using MSE:

$$\begin{array}{c} L\left(x,x^{\prime }\right)={\left\|x-x^{\prime }\right\|}^{2} \end{array}$$ (9)

This loss function measures the squared difference between each element in *X* and *X*′. The final encoded representation *X*_encoded_ that is used for further processing (like feature selection or classification) can be represented as:

$$\begin{array}{c} X_{\text{encoded}}=f_{3}\left(W_{3}f_{2}\left(W_{2}f_{1}\left(W_{1}X+b_{1}\right)+b_{2}\right)+b_{3}\right) \end{array}$$ (10)

This composite function represents the output of the encoder, which is a function of input *X* and the parameters of the three layers.

Proper tuning of hyperparameters, such as depth, layer sizes, activations, batch size, epochs, and learning rate, is crucial for optimizing the SAE data compression and reconstruction capability. Random search provides an efficient mechanism for sampling hyperparameter sets randomly from predefined ranges to identify high-performance configurations with limited computational overhead.

The optimized SAE provides a compressed nonlinear representation of the input for dimensionality reduction. Reduced encoding retains the most informative attributes and relationships in a lower-dimensional space that is suitable for resource-constrained IoT environments.

#### 3.2.2 CatBoost for predictive feature selection

We utilized CatBoost for efficient feature selection in our machine learning pipeline. CatBoost is an open-source gradient-boosting library developed by Yandex that is known to handle categorical variables and achieve state-of-the-art performance [61]. A key advantage of CatBoost is its built-in feature-selection functionality. As CatBoost trains the models, it assigns importance to each feature, based on the predictive value of the object variable. We used this method to identify and select the most significant features. By removing low-importance features, we simplified our models, reduced overfitting, and improved performance. CatBoost provides an automated method for selecting the most informative features and discarding the uninformative ones. This allows us to build more accurate and interpretable models by focusing on the features that truly drive our machine-learning task.

Although SAEs reduce the number of dimensions, some redundant and irrelevant features may persist. To refine the feature space, a CatBoost model is trained on SAE-encoded features to predict the target variable. CatBoost is a high-performance gradient-boosting algorithm that is well-suited for feature selection. The steps involve:

- Training a CatBoost model on the SAE encoded features.
- Computing feature importance scores.
- Sorting features by importance ranking.
- Selecting the top N most predictive features.

**Fig 2:**
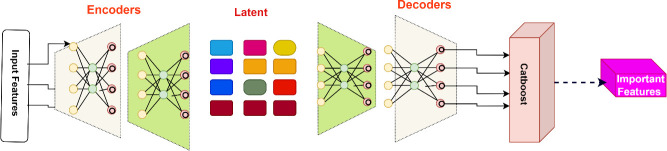
Feature engineering pipeline using stacked autoencoders and CatBoost: The pipeline starts with the input data, which is processed by a stacked autoencoder for nonlinear dimensionality reduction. The encoded features from the autoencoder are then input into CatBoost, which computes feature importance scores and selects the top N most predictive features.

The integrated approach extracts optimized and compact feature subsets tailored to the learning task, as shown in Fig 2. This combines the strengths of unsupervised nonlinear SAE feature reduction with supervised CatBoost predictive feature selection based on the importance ranking. The integrated approach extracts optimized and compact feature subsets tailored to the learning task.

The compact informative features also align with the narrow memory and processing requirements of IoT and edge devices. The integrated SAE-CatBoost pipeline provides an end-to-end feature-engineering solution to derive optimized feature representations from high-dimensional network traffic data for efficient intrusion detection in IoT environments.

After dimensionality reduction by SAE, CatBoost was used to perform feature selection on the encoded features *X*_encoded_:

CatBoost(X_encoded, y)

The features were sorted based on their importance scores, and the top N features were selected:

X_selected = select_top(X_encoded, N)

CatBoostClassifier.fit(X_train_encoded, y_train)

#### 3.2.3 Feature-importance computation

After training, the feature importance scores were computed using the CatBoost model, which gives us a score for each feature:

$$\begin{array}{c} \text{Importance}\left(i\right)=\text{Importance}\left(x_{i}\right) \end{array}$$ (11)

Where Importance(*i*) represents the importance score of the *x*_*i*_ features. The selected features for the AWID Binary, ranked by their importance, begin with F8 at the top, followed by F3, F4, F16, and F0, which rank in the top five. The next set of significant features includes F27, F31, F17, and F20, with F25 completing the top ten. As we move down the list, F11, F6, F29, F24, and F12 offer considerable value. Other notable features included F19, F7, F28, F26, and F14. The list continues with F2, F21, F1, and F5 and is rounded off with F13, F23, F18, F9, F22, F15, F10, and F30, showing a diverse range of factors considered in the AWID Binary dataset, as shown in Table 1.

**Table 1:** Selected features for the AWID dataset.

Binary		Multi	
Feature	Importance	Feature	Importance
F8	14.272595	F18	12.992755
F3	9.232790	F10	8.758666
F4	8.580130	F14	7.340345
F16	7.804091	F24	7.034608
F27	5.830682	F4	5.177741
F31	5.708804	F25	4.997708
F17	5.618724	F27	4.308031
F20	5.546029	F16	3.927319
F25	4.573633	F7	3.721938
F11	3.754997	F8	3.662475
F6	3.622543	F22	2.997933
F29	2.637549	F28	2.976423
F24	2.574034	F23	2.910958
F12	2.283176	F13	2.889712
F19	2.260098	F19	2.659953
F7	1.668862	F6	2.560386
F28	1.453495	F30	2.552735
F26	1.182538	F12	2.423462
F2	1.013080	F31	2.125745
F21	0.902356	F17	1.981360
F1	0.857318	F20	1.229678
F5	0.551093	F15	0.771426
F13	0.543342	F1	0.579557
F23	0.356229	F3	0.381941
F18	0.0317352	F29	0.224604
F9	0.030222	F11	0.046443
F22	0.02400	F21	0.017679
F15	0.021000	F9	0.0110000
F10	0.019100		
F30	0.01200		

This table displays the selected features for the AWID dataset in both binary and multi-classification contexts, along with their corresponding importance values.

For the AWID features in the multi-dataset, the selected features, totalling 30, were arranged in order of importance. The list begins with F18 leading the pack, followed by F10, and F14. F24 and F2 were also highly ranked and rounded out among the top five. The dataset continued to leverage the significance of F4, F25, F27, F16, and F7, with F8 being slightly behind. Additional features, including F22, F28, F23, F13, and F19, provided valuable information. The series continued with F6, F30, F12, and F0, each of which added depth to the model. Concluding the feature set, F31, F17, F20, F15, F1, F3, F29, F11, F21, and F9, despite their lower ranks, play a part in the model’s foretelling capacity, showcasing the broad nature of the AWID Features on Multi classification.

For the UNSWB15 Binary dataset, 21 features were carefully chosen for their unique contribution to the model’s performance. The arrangement started with F1, taking the prime spot, followed by F2, F3, F4, and F5, making up the foremost quintet. The selection proceeds with pivotal features such as F6, F7, F8, F9, and F10, each playing a crucial role. The enumeration continued with F11, F12, F13, F14, F15, F16, F17, F18, F19, F20, and F21, collectively adding depth, variety, and acuity to the dataset and illustrating the extensive and insightful selection of features for the UNSWB15 Binary dataset.

For UNSWB15 multi-features, 26 features were selected based on their importance. The list begins with F13 at the forefront, followed by F1 and F15. F29 and F12 also held significant ranks, completing in the top five. The dataset includes valuable inputs from F4, F23, F34, F9, and F31. Additional layers of complexity and insight were provided by F26, F37, F32, F20, F18, F10, F0, F5, F25, F21, F14, F19, F11, F7, F36, and F30, each contributing to the model’s predictive capabilities, demonstrating a comprehensive selection of features for the UNSWB15 multi-dataset as described in Table 2.

**Table 2:** Selected features for the UNSWB15 dataset.

Binary		Multi	
Feature	Importance	Feature	Importance
F2	2.769314	F1	8.368968
F3	1.921917	F15	7.977230
F4	1.706085	F29	7.602685
F5	4.063683	F12	6.111354
F6	4.219298	F4	5.729292
F7	0.904238	F23	5.039764
F8	1.893601	F34	4.219298
F9	2.315674	F9	4.063683
F10	0.907848	F31	4.017140
F11	7.602685	F26	3.525911
F12	10.170015	F37	3.254313
F13	8.368968	F32	2.769314
F14	6.111354	F20	2.672921
F15	5.729292	F18	2.315674
F16	5.039764	F10	1.921917
F17	1.748686	F0	1.893601
F18	1.738093	F5	1.748686
F19	1.059278	F25	1.738093
F20	4.017140	F21	1.706085
F21	2.672921	F14	1.352462
F22	1.048385	F19	1.059278
F23	0.925848	F11	1.048385
F24	7.977230	F7	0.925848
F25	3.525911	F36	0.907848
F26	4.219298	F30	0.904238

This table displays the selected features for the UNSWB15 dataset in both binary and multi-classification contexts, along with their corresponding importance values.

For the KDD Binary dataset, a carefully curated set of 25 features is selected based on their importance. Topping the list is F26 ‘srv_serror_rate,’ demonstrating paramount significance with the highest importance score. Not far behind is F32 ‘dst_host_count,’ cementing its critical role in the dataset. F10 ‘hot’ follows suit as a key indicator, with F35 ‘dst_host_diff_srv_rate’ and F12 ‘logged_in’ rounding out the top five, each bringing substantial weight to the predictive model. Continuing down the list, F5 ‘src_bytes’ and F17 ‘num_file_creations’ emerge as notable features, along with F2 ‘protocol_type’ and F18 ‘num_shells’, each contributing their unique insights. F1 ‘duration’ seals the top ten, representing a vital aspect of the data. The mid-range of the importance spectrum includes features such as F36 ‘dst_host_same_src_port_rate,’ F8 ‘wrong_fragment,’ and F9 ‘urgent,’ all adding valuable dimensions to the analysis. Features such as F23 ‘count,’ F30 ‘diff_srv_rate,’ and F29 ‘same_srv_rate’ enhance the model further, while F24 ‘srv_count’ and F7 ‘land’

For the KDD Multi-dataset, 25 features were carefully selected based on their significance scores. The lineup began with F37, which marked its prominence as the most influential feature. This is closely followed by F4, which has substantial weight in the model. F31 also has a notable appearance, securing a key position in the lineup. F22 and F2 were also pivotal, rounding out the top five most influential features, and the selection continued with F26 and F12, each providing critical insights into the dataset. F28, F36, and F7 further enriched the model and highlighted their importance. The array was complemented by F41, F8, F27, and F30, each of which provided valuable perspectives. F1, representing a temporal aspect, along with F35, F24, and F32, contribute significantly to the dataset’s complexity. Further down the list, F23 and F34 add depth to the analysis, while F33 provides essential data interpretation. The set included F29, F38, F3, and F25, each offering unique contributions and ensuring a comprehensive approach to the feature selection process for the KDD Multi-dataset. By combining the original and encoded feature analyses, a holistic perspective was obtained to judiciously leverage the selected features during the model development, as shown in Tables 1–3.

**Table 3:** Selected features for the KDD dataset.

Binary		Multi	
Feature	Importance	Feature	Importance
f32	13.006572	f4	5.391645
f10	7.074625	f31	4.722989
f35	6.278346	f22	4.184788
f12	5.711010	f2	4.098703
f5	4.691332	f26	3.814086
f17	3.717627	f12	3.699748
f2	3.532182	f28	3.637082
f18	3.053878	f36	3.611243
f1	3.030462	f7	3.518334
f36	2.545313	f41	3.254036
f8	2.302747	f8	3.170991
f9	2.215344	f27	2.930900
f23	2.215306	f30	2.877749
f30	2.046981	f1	2.844795
f29	2.014834	f35	2.794198
f24	1.915516	f24	2.742301
f7	1.600220	f32	2.701303
f22	1.435186	f23	2.698559
f36	1.384915	f34	2.671701
f12	1.303449	f6	2.480610
f10	1.075029	f29	2.426426
f35	1.009861	f38	2.390946
f34	1.004063	f3	2.299040
f31	0.989742	f25	2.099261

This table describes the selected features for the KDD dataset in both binary and multi-classification contexts, along with their corresponding importance values.

### 3.3 The ensemble model architecture

Transformer neural networks (TNN) were first introduced in 2017 for sequence modelling tasks in natural language processing (NLP) [62]. Although off-the-shelf TNN models, such as BERT and GPT, have achieved strong results on NLP problems, they may not be directly transferred to intrusion detection owing to differences in input data types and objectives. While NLP TNN models process sequences of words or tokens, intrusion detection systems process sequential network traffic and event data. Hence, this study proposes a customized TNN architecture tailored to the unique requirements of network intrusion detection.

A key advantage of TNN is its self-attention mechanism, which can capture the dependencies across a sequence. This allows a TNN-based intrusion detection model to analyze network traffic patterns and identify anomalies indicative of threats. Although other neural architectures exist for anomaly detection, TNN’s strengths in modelling complex sequential relationships make it well-suited for security tasks.

**Fig 3:**
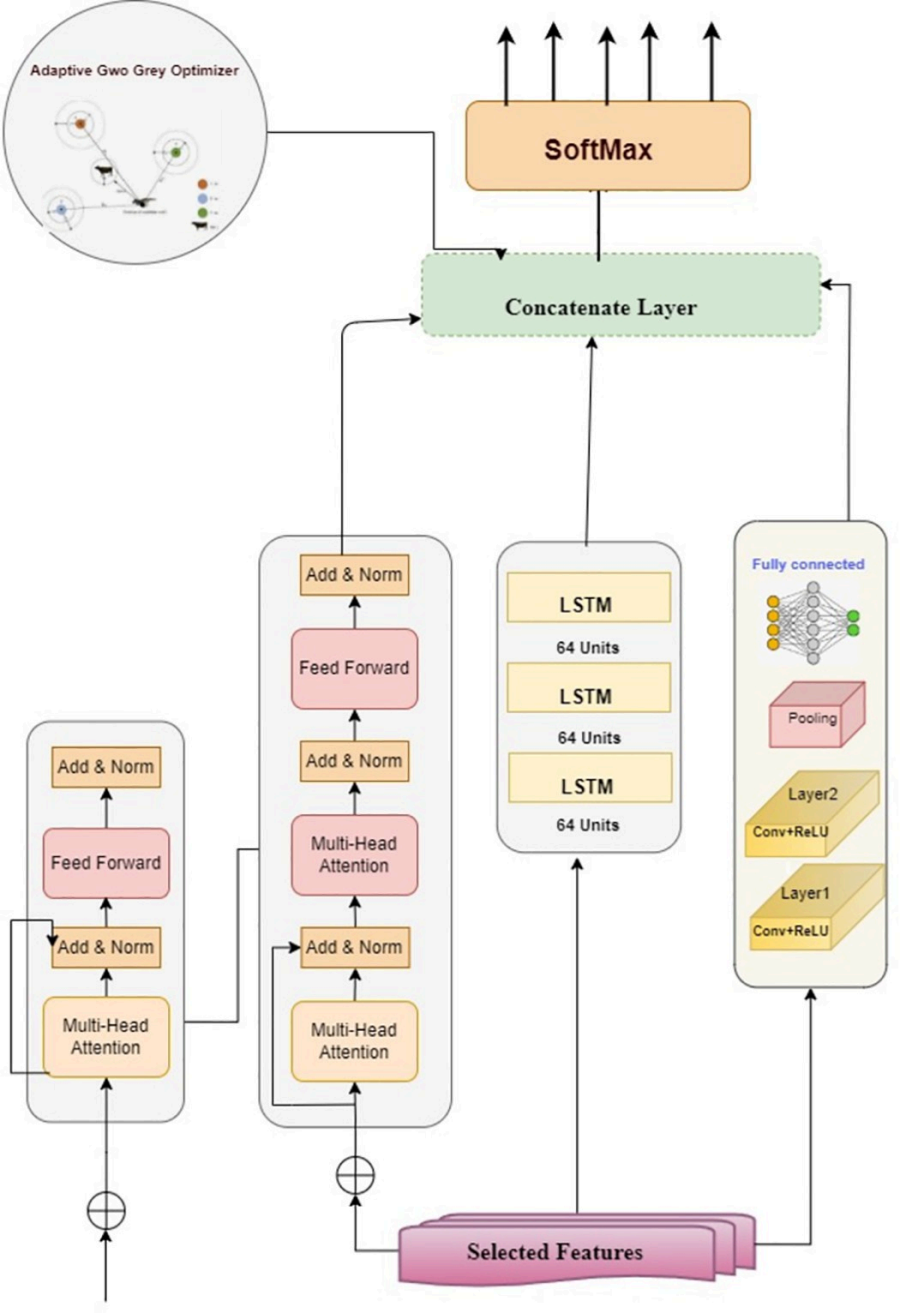
Ensemble neural network architecture integrating transformers, CNNs, and LSTMs. It shows the parallel application of the Transformer, CNN, and LSTM branches to the input sequence data. Their outputs were concatenated to ensemble global, local, and temporal patterns in the data for robust sequence modelling and intrusion detection.

Advanced neural architectures, such as Transformers, CNNs, and LSTMs, have achieved state-of-the-art results across various sequence-modelling tasks. Transformers utilize multihead self-attention to analyze the interactions between all positions in a sequence. CNNs leverage convolutional filters to recognize local spatial patterns. LSTMs employ gated memory cells to capture long-range dependencies. This study proposes a novel ensemble model integrating Transformers, CNNs, and LSTMs to synergistically combine their complementary strengths for robust network intrusion detection, as shown in Fig 3.

#### 3.3.1 Transformer

The Transformer revolutionized sequence modelling through self-attention. Rather than processing a sequence recursively like RNNs, self-attention identifies the relationships between all input positions in parallel. The Transformer block constitutes an integral component within the overall architectural framework and is optimized for sequential modelling tasks. Leveraging the mechanistic construct of multiheaded self-attention, each transformer block enables parameterized weighting of relevance across input tokens. We instantiate the embedding layer with dimensionality aligned to the raw input representation to enable the initial projection of the inputs. As elucidated in the schematic, we configured each transformer block with eight parallel attention heads, thus allowing the capture of diverse latent interactomics. Additionally, we designed the feedforward subcomponent with 64 hidden units and ReLU nonlinearity to induce representational depth. To encourage robust optimization dynamics, we incorporate dropout regularization after each subcomponent with a rate tuned to 0.3, alongside LayerNorm for sufficient conditioning of internal representations. The learning rate annealing schedule applies an exponential decay methodology with an initial value of 0.01 decaying by a ratio of 0.9 every 10,000 update steps. For parameter optimization, we selected the Adam variant for typical considerations of the computational efficiency and memory footprint. In addition, the model first defines a Keras input layer to match the shape of the input data. It then stacks three transformer blocks with configurations for the embedding dimension, number of heads (8), and feedforward layer dimensionality. Global average pooling was applied to the transformer output to aggregate the representations across tokens.

The attention mechanism is defined mathematically as:

$$\begin{array}{c} \text{Attention}\left(Q,K,V\right)=\text{softmax}(\frac{QK^{T}}{\sqrt{d_{k}}})V \end{array}$$ (12)

where *Q*, *K*, and *V* are the query, key, and value matrices representing the embedded inputs, respectively. The dot product score similarities between queries and keys and values are aggregated based on attention weights.

Multihead attention projects inputs into *h* heads and concatenates their results:

$$\begin{array}{c} \text{MultiHead}\left(Q,K,V\right)=\text{Concat}\left(head_{1},\ldots ,head_{h}\right)W^{O} \end{array}$$ (13)

where

$$\begin{array}{c} head_{i}=\text{Attention}\left(QW_{i}^{Q},KW_{i}^{K},VW_{i}^{V}\right) \end{array}$$ (14)

This allows for the modelling of different types of interactions. Residual connections and layer normalization stabilize training:

$$\begin{array}{c} \text{LayerNorm}(x+\text{Sublayer}(x)) \end{array}$$ (15)

Stacking Transformer blocks enables learning of hierarchical representations. Self-attention provides global context modeling.

#### 3.3.2 CNN

Convolutional neural networks apply local filters across inputs to extract spatial features. The 1D convolutions for sequence data are computed as follows:

$$\begin{array}{c} c_{ij}=b+\sum_{k}W_{ijk}\cdot x_{i-k+1:i+k-1} \end{array}$$ (16)

where, Wk is the filter weight, b is the bias, and xi:i+k-1 is the input patch. Stacking convolutional layers enable the learning of hierarchical local motifs. Batch normalization and nonlinearities aid in the training. CNNs have achieved state-of-the-art results in computer vision applications and in many fields.

#### 3.3.3 LSTM

Long Short-Term Memory (LSTM) networks [37]. are a type of RNN well suited for sequence modeling. The LSTM cells contain input, output, and forget gates.

$$\begin{array}{c} i_{t}=\sigma \left(W_{xi}x_{t}+W_{hi}h_{t-1}+b_{i}\right) \end{array}$$ (17)

$$\begin{array}{c} f_{t}=\sigma \left(W_{xf}x_{t}+W_{hf}h_{t-1}+b_{f}\right) \end{array}$$ (18)

$$\begin{array}{c} o_{t}=\sigma \left(W_{xo}x_{t}+W_{ho}h_{t-1}+b_{o}\right) \end{array}$$ (19)

The gates manipulation the information stream in the cell state. This architecture selectively remembers patterns in sequences even over long durations. The LSTM model consists of a two-layer network designed for sequence modeling. The first layer is an LSTM layer with 64 units that returns sequences, meaning that it provides an output for each input in the sequence. This is crucial when stacking LSTM layers, as it ensures that the second LSTM layer receives a three-dimensional sequence input. The second LSTM layer also has 64 units but does not return sequences; thus, it only provides an output for the last input in the sequence, effectively condensing the sequence into a single two-dimensional vector. Each LSTM unit comprises input, output, and forget gates that control the flow of information in the cell state. This allows the LSTM to selectively remember patterns in sequences over extended durations, making it particularly effective for sequence-modeling tasks.

#### 3.3.4 Ensemble integration

The Transformer, CNN, and LSTM branches were applied parallel to the inputs. Their outputs are concatenated to the ensemble global, local, and temporal features as follows:

$$\begin{array}{c} h=[\text{Transformer}(x);\text{CNN}(x);\text{LSTM}(x)] \end{array}$$ (20)

The model was trained end-to-end, supporting inter-branch feature sharing and adaptation. This novel hybrid design leverages the complementary strengths of transformers, CNNs, and LSTMs for robust network sequence modelling. The ensemble architecture provided multiscope analysis capabilities.

### 3.4 Optimization

Adaptive Grey Wolf Optimization (AGWO) is a nature-inspired metaheuristic algorithm that takes inspiration from the hunting behavior of grey wolves in the wild [63]. Grey wolves have a strict social hierarchy and hunting strategy involving tracking, encircling, and attacking prey.

The AGWO technique translates these behaviors into optimization procedures. The fittest gray wolf pack was identified based on the accuracy of the ensemble model on a validation dataset. The alpha wolf guides the optimization search. Encircling prey translates into exploring the solution space around the current optimal hyperparameter values.Attacking prey corresponds to rapidly exploding promising areas for improved solutions. In addition, the AGWO implements adaptive mechanisms to prevent suboptimal convergence. Varying the exploration-exploitation tradeoff over iterations. Randomize the search vectors to maintain diversity. Gradually reducing the exploration area. These adaptive enhancements prevent the algorithm from becoming trapped in the local optima. By mimicking the hunting behaviours and mechanisms of grey wolves, AGWO provides an effective bio-inspired approach to tune hyperparameters, such as learning rate, neural architecture dimensions, regularization strength, and other facets of the Transformer-CNN-LSTM ensemble model. The goal is to maximize network intrusion detection tasks’ accuracy and generalization performance. The model integrates self-attention-based transformers, local feature-extracting CNNs, and long-range LSTM networks to provide complementary global, local, and temporal modeling capabilities. However, finding the correct combination of hyperparameters, such as learning rates, layer sizes, and regularization for each branch, is challenging. AGWO provides an effective bio-inspired optimization technique. AGWO adapts the hunting, encircling, and attacking mechanisms of gray wolves to guide hyperparameter values toward optimal settings. We developed an extremely optimised hybrid model by applying AGWO to tune the hyperparameters of the Transformer, CNN, and LSTM branches in our ensemble. This provides the accuracy and robustness benefits of the multiscope Transformer-CNN-LSTM architecture, which is further boosted by the adaptive optimization power of AGWO.

**Algorithm 1** AGWO for Ensemble Neural Network Model Optimization

1: Generate initial grey wolf positions *Y*(*i*) for *i* = 1, 2, …, *n*

2: Initialize vectors *a*, *A*, and *C*

3: **for** each wolf *Y*(*i*) **do**

4:  Create an ensemble of models using the parameters from *Y*(*i*)

5:  Evaluate the fitness of each ensemble (accuracy, loss, etc.)

6: **end for**

7: *Y*(*α*) = Ensemble with best performance

8: *Y*(*β*) = Ensemble with second-best performance

9: *Y*(*δ*) = Ensemble with third-best performance

10: Set iteration counter *I* = 1

11: **repeat**

12:  **for** each grey wolf *Y*(*i*) **do**

13:   Update the position of *Y*(*i*) (parameters for the ensemble)

14:   Create a new ensemble of models with updated parameters

15:  **end for**

16:  Evaluate the fitness of all new ensembles

17:  Update the positions of *Y*(*α*), *Y*(*β*), and *Y*(*δ*) based on new fitnesses

18:  Update vectors *a*, *A*, and *C*

19:  $a=2-I\times (\frac{2}{\text{max\_iterations}\times \sqrt{I+1}})$

20:  **for** each dimension **do**

21:   Update *A* and *C* using random values

22:   Calculate new positions based on *Y*(*α*), *Y*(*β*), and *Y*(*δ*)

23:  **end for**

24:  *I* = *I* + 1

25: **until** *I* ≥ max_iterations

26: Output *Y*(*α*) as the best solution (optimal ensemble parameters)

**Fig 4:**
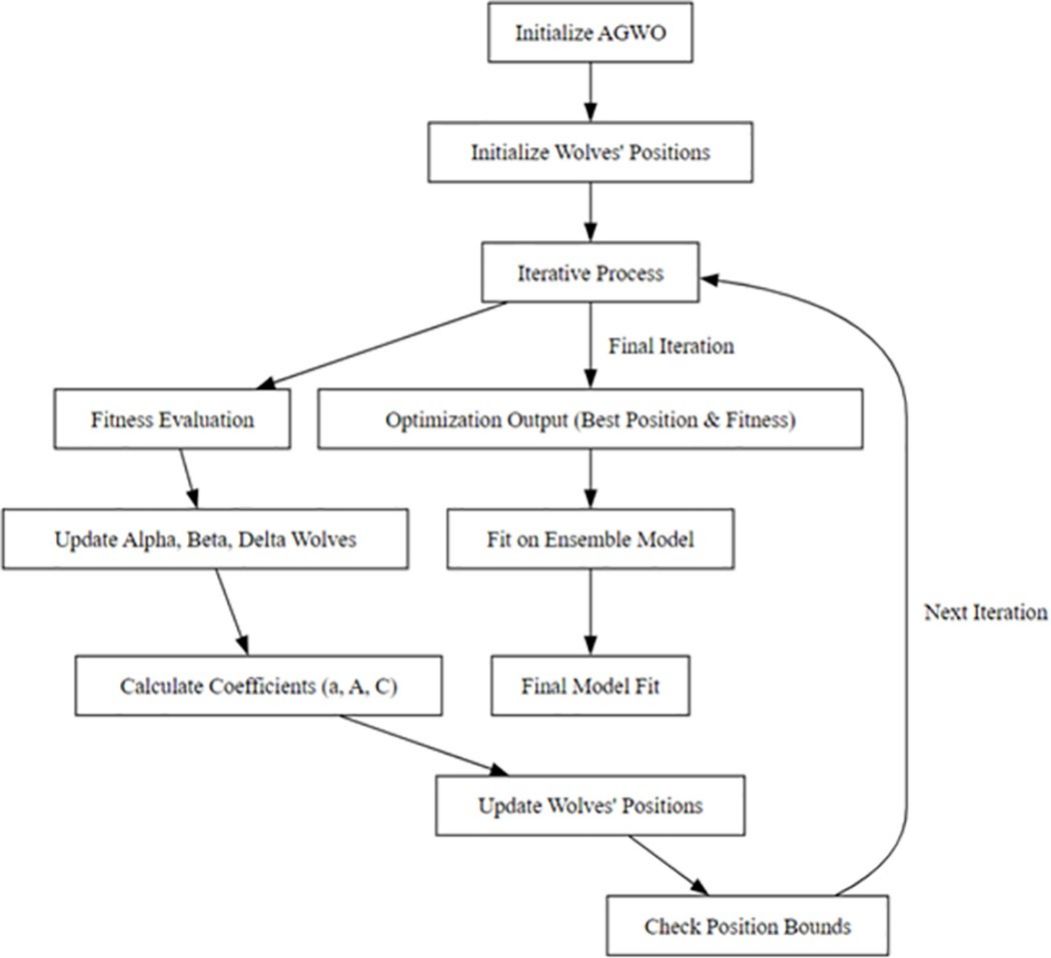
A flowchart diagram of the iterative optimization process used by the adaptive grey wolf optimization algorithm to tune the hyperparameters of the ensemble model. It depicts the steps of initializing the grey wolf population, evaluating ensemble models, updating wolf positions, re-evaluating models, and adaptively adjusting the optimization vectors to hone in on the optimal hyperparameters.

The AGWO algorithm initializes a population of grey wolves, where each wolf’s position represents a set of parameters for an ensemble of neural network models. An ensemble of models was created for each set of parameters, and its performance was evaluated based on metrics such as accuracy and loss. The top three ensembles were identified in order to establish a hierarchy. The main loop then iteratively updates the wolf positions (parameters) and reevaluates the ensembles. For each updated position, a new ensemble was created and evaluated. The vectors A, and C are adjusted adaptively to guide the search process towards the optimal parameters. The main loop continues for a predefined number of iterations. Finally, the parameters leading to the best-performing ensemble were the output. AGWO leverages adaptive biologically inspired operators to optimize the hyperparameters of an ensemble model by iteratively creating and assessing ensembles and honing optimal parameters as depicted in Fig 4. This provides an effective technique for tuning and maximizing the performance of an integrated neural network model.

## 4 Experimental setup

The proposed techniques were implemented leveraging TensorFlow 2.8.0 with the Keras API for constructing, training, and evaluating specialized neural network architectures, including SAEs, CNN, LSTM Models, and Transformer Networks tailored to resource-constrained edge devices. SciKit-Learn 1.0.2 enabled optimized data preprocessing pipelines, Pandas 1.3.5 facilitated dataset manipulation, and Matplotlib 3.5.1 assisted analysis and visualization. The experiments used an Intel Core i7-12700K processor with 32GB DDR5 memory and an Nvidia RTX 3090 GPU with 1TB PCIe 4.0 NVMe solid-state storage to enable efficient iteration and experimentation with complex deep learning topologies, synergizing model, data, and pipeline parallelism to accelerate training. This section demonstrates the performance of the proposed technique for intrusion detection using stacked autoencoders (SAE) for feature dimensionality reduction and CatBoost for feature selection. The SAE performs nonlinear encoding to reduce the dimensionality of input features. CatBoost is then trained on these encoded features to compute the importance scores and select the most predictive subset of features. The reduced encoded feature representations are input into an ensemble model comprising Transformers, CNNs, and LSTMs for intrusion detection sequence modelling. AGWO was used to optimize the hyperparameters of the ensemble model to maximize the detection performance. The objective is to achieve effective intrusion detection with optimal classification performance by: Applying SAE for nonlinear dimensionality reduction Using CatBoost for predictive feature selection Feeding optimized features into the ensemble model Tuning the ensemble hyperparameters with AGWO Table 1 shows the performance of the proposed technique on benchmark datasets compared to the current methods. The results demonstrated favourable accuracy, F1-score, and ROC-AUC, indicating the efficacy of this approach.

### 4.1 Experimental datasets

This research utilized three standard network intrusion detection benchmark datasets to rigorously evaluate the proposed system. NSL-KDD: This dataset encompasses network traffic data with both regular connections and intrusion attacks [64]. The training and testing subsets were comprised of 125,973 and 22,544 samples, respectively. It has 41 features describing each network flow related to the protocol, statistics, content, etc. Binary classification for detecting attack flows and multi-class modelling of specific intrusion types were investigated. For the binary classification task of detecting attack vs. normal flows, 25 key features were selected from the 41 total features using the proposed SAE and CatBoost pipelines. For multi-class modelling with five classes (Normal, DoS, Probe, R2L, U2R), 30 predictive features were extracted from the 41 available features. UNSW-NB15: This more recent dataset emulates a hybrid enterprise deployment with 49 features across flow, basic, content, and time categories [65]. The training and testing sets consisted of 175,341 and 82,332 samples, respectively. It covers 10 classes, including normal and nine contemporary attack types, such as worms and malware, and exploits categories tailored to current network threats. The multi-class classification was performed to discriminate between the attack varieties. For the binary task of identifying malicious connections, 21 features were selected from the total 49 features. For multi-class modelling across the 10 classes, 26 informative features were derived using the feature engineering workflow. AWID: The Aegean Wi-Fi Intrusion Dataset focuses specifically on 802.11 wireless networks with major protocols [66]. It contains over 150 attributes that capture different properties of wireless traffic. The dataset includes normal data and flooding, impersonation, and injection attacks targeting the wireless environment. A total of 1,797,575 samples were collected across the four classes. Binary classification was used to distinguish between benign and malignant network flow. For the binary classification task, 30 features were selected from more than 150 attributes. For the AWID multi-class task, 29 predictive features were selected from over 150 total features using the proposed SAE and CatBoost pipelines. Together, these datasets enable assessment across traditional networks, modern hybrid enterprise deployments, and wireless-specific threats. The range of features, attack types, and environments make the datasets complementary and suitable for holistic evaluation. Optimized and compact feature subsets were derived for each dataset and task using the proposed integrated SAE and CatBoost approach. This has enabled the development of specialized models focused on the most predictive attributes for enhanced performance.

## 5 Results

### 5.1 Stacked autoencoder architecture

**Fig 5:**
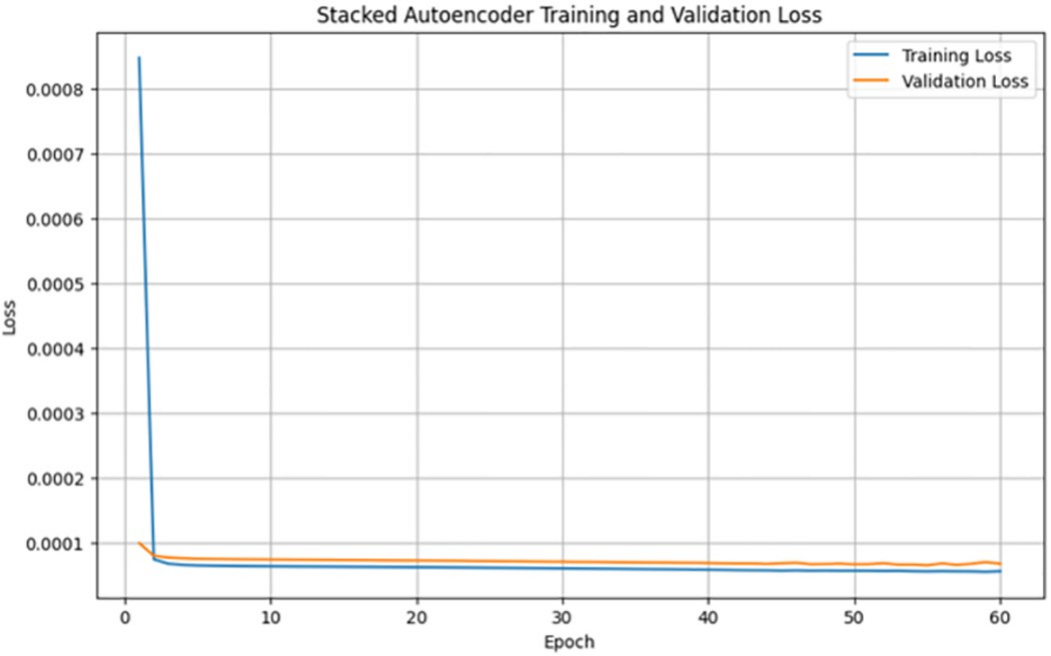
Loss reduction over epochs in stacked autoencoder training vs validation on NLS-KDD for multi-classification: It illustrates the training and validation loss over epochs during the training of a stacked autoencoder on the NLS-KDD dataset for multi-classification. The blue line represents the training loss, and the orange line represents the validation loss. Both the losses decreased over time, indicating the effectiveness of the training process.

**Fig 6:**
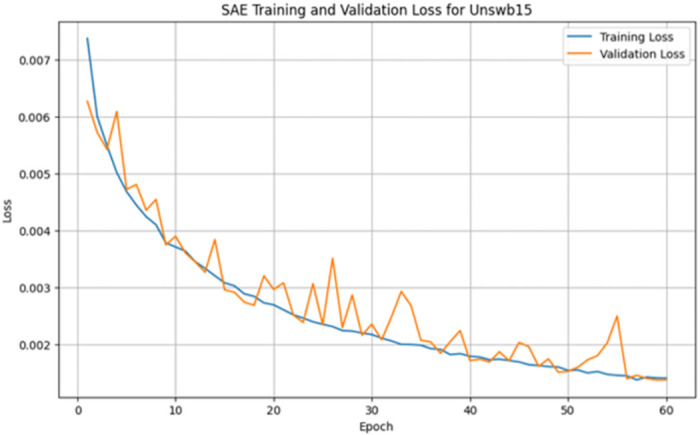
Loss reduction over epochs in stacked autoencoder training vs validation on Unswb15 for multi-classification.

**Fig 7:**
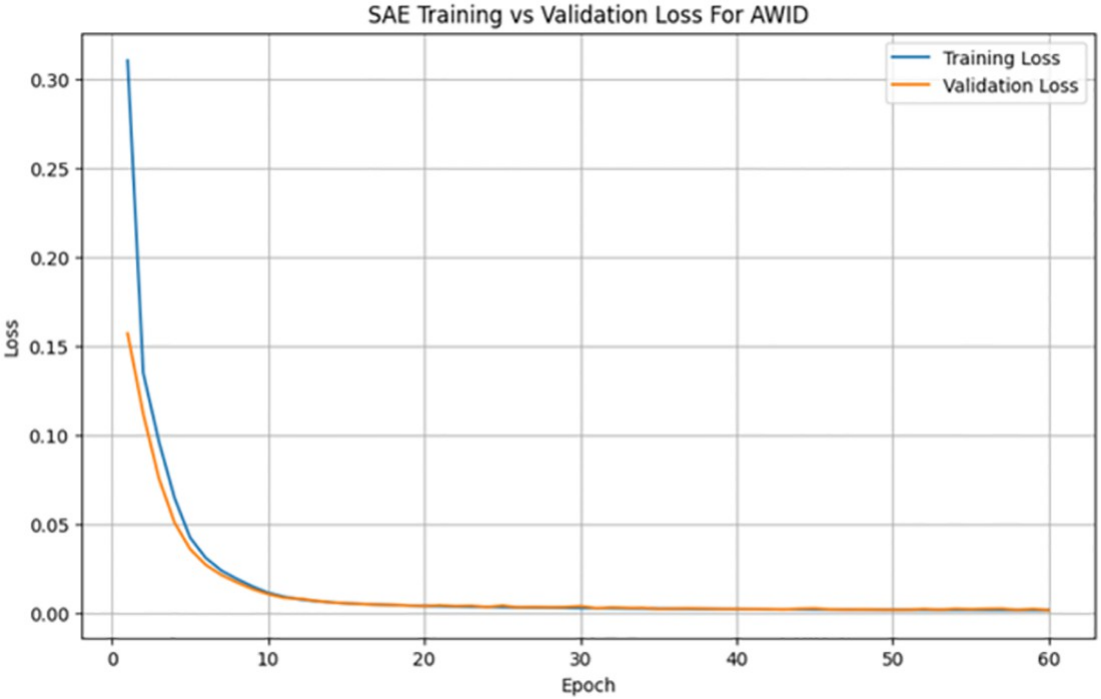
Loss reduction over epochs in stacked autoencoder training vs validation on AWID for multi-classification.

A stacked autoencoder (SAE) is an unsupervised artificial neural network used for nonlinear dimensionality reduction and is often applied for IDS in network traffic data. The input data consists of network traffic datasets that first undergo preprocessing to extract numeric input features (denoted by X) and categorical label targets (denoted by y). The popular Python machine learning library sci-kit-learn provides tools for handling data wrangling and preprocessing tasks. Specifically, the data were split in an 80/20 ratio into training and testing sets. This allowed the models to be trained on a larger training set and then evaluated on the unseen testing set. Additionally, min-max scaling, a feature normalization technique, is used to normalize the range of input features to be between 0 and 1. This preprocessing step is crucial because neural networks learn and perform better when the input data features are scaled similarly. The input dimension for the SAE matches that of the scaled training data matrix, ‘X_train_scaled’. The architecture consists of three encoding layers that incrementally compress the input features into a reduced lower-dimensional, followed by three symmetric decoding layers that mirror the encodings to reconstruct the original high-dimensional input feature space at the output. This symmetrical autoencoder structure forces the model to learn efficient data encoding to produce an accurate reconstruction. The encoding layers utilize the Rectified Linear Unit (ReLU) activation function to introduce nonlinearity, which simply returns the input value itself if it is positive; otherwise, it outputs zero for negative inputs. In contrast, the decoding layers mirror the encoding dimensions in the reverse order. The SAE is implemented in Keras using dense layers for the encoders and decoders. The input layer size matched the number of input features in the preprocessed dataset (41 for NSL-KDD). This is encoded down to a smaller bottleneck layer of size 16, which retains only the most salient information, and the encoding layers use the rectified linear unit (ReLU) activation function and He normal kernel initializer. L2 regularization, including a strength of 0.001, was used on each encoding layer to prevent overfitting. The decoding layers mirror the encodings in reverse order to reconstruct the input, and the autoencoder is trained for 60 epochs using the Adam optimizer by a learning rate of 0.001 to reduce the MSE between the input and reconstructed output. The batch size was 256 samples, and the validation data were used to track overfitting. Once trained, the 16-node encoded bottleneck was used as the reduced feature input for the ensemble classifier, which involved a grid search over encoder sizes [32, 64, 128, 256], bottleneck size [8, 16, 32], regularization strength [0.0, 0.001, 0.01], learning rate [0.0001, 0.001], and epochs [50, 100, 150] with 30 trials per configuration to determine the optimal SAE parameters. SAEs effectively compress high-dimensional input spaces while retaining the most informative attributes, as validated through multiple reconstruction error metrics. On the NSL-KDD dataset [64], the mean squared errors averaged 0.021, whereas the mean absolute errors remained under 0.071 across both binary and multi-class tasks (Table 4). The close-to-unity cosine similarity of 0.999 further indicates that the SAE accurately captures intrinsic data patterns. The results on the more contemporary UNSW-NB15 dataset [65] in Table 5 echo the strong reconstruction capability of the SAE, with mean squared errors of 0.005 and 0.009 for multi-class and binary activities, respectively. The wireless-focused AWID dataset [66] evaluations in Table 6 produced comparable outcomes, confirming that the SAE consistently models the essence of diverse input features across domains. Overall, dimensionality reduction preserves salient properties for high-fidelity feature extraction tailored to the fog nodes. Figs 5–7 show the loss reduction over epochs during the SAE training and validation on the NSL-KDD, UNSW-NB15, and AWID datasets.

**Table 4:** Results of binary and multi-classification on NLS KDD using SAE.

NLS KDD	Classification Type	Mean Squared Error	Mean Absolute Error	Cosine Similarity	PCC
	Multi-Class	0.0214	0.0718	0.999	0.99
	Binary	0.02148	0.071	0.999	0.99

This table depicted the results for binary and multi-class classification on the NLS KDD dataset using SAE, detailing metrics like Mean Squared Error, Mean Absolute Error, Cosine Similarity, and Pearson Correlation Coefficient.

**Table 5:** Results of binary and multi-classification on Unswb15 using SAE.

unswb15	Classification Type	Mean Squared Error	Mean Absolute Error	Cosine Similarity	PCC
	Multi-Class	0.0050	0.028	0.997	0.997
	Binary	0.0090	0.044	0.995	0.995

This table displays the results for binary and multi-class classification on the Unswb15 dataset using SAE, detailing metrics like Mean Squared Error, Mean Absolute Error, Cosine Similarity, and Pearson Correlation Coefficient.

**Table 6:** Results of binary and multi-classification on AWID using SAE.

AWID	Classification Type	Mean Squared Error	Mean Absolute Error	Cosine Similarity	PCC
	Multi-Class	0.00420	0.029	0.998	0.998
	Binary	0.0001	0.0020	0.999	0.999

This table provides detailed metrics for binary and multi-class classification on the AWID dataset using SAE, including Mean Squared Error, Mean Absolute Error, Cosine Similarity, and Pearson Correlation Coefficient.

### 5.2 CatBoost feature selection process

CatBoost, an ensemble learning algorithm based on gradient boosting, is a powerful tool for feature selection and predictive modelling. This section delves into the intricacies of CatBoost, elucidating its input, output, and hyperparameters as well as its significance in feature selection. The encoded dataset, ‘*X*_*train*_*encoded*’, was then provided as an input to the CatBoost model for feature selection. CatBoost evaluates the importance of each feature in the encoded data based on its contribution to the predictive performance of the model.

#### 5.2.1 Evaluating feature selection

For CatBoost, the following key hyperparameters governing the predictive feature selection were tuned:

1. Number of trees: Tested in the range of [100, 500] in increments of 100.
2. Maximum tree depth: Values from 5 to 8 were explored.
3. Learning rate: Varied over intervals [0.025, 0.1].
4. L2 leaf regularization: Tested lambda from [1, 5].

The CatBoost model underwent training with an optimal count of trees and depths. A learning rate set at 0.05, coupled with an L2 regularization strength of 3, yielded the most effective predictive outcome as per cross-validation. This specific setup was employed to determine feature significance and to choose the superior subsets. To assess the effectiveness of the selected features, the original dataset was filtered to retain only the top N features identified using CatBoost. This filtered dataset, with only the most important features, is denoted by

X_train_selected, and the model is then trained on

X_train_selected and tested on the corresponding filtered test set

X_test_selected. Comparing the performance of the model with all the original features quantifies the impact of the feature selection. CatBoost evaluates the importance of each feature in the encoded data based on its contribution to the predictive performance of the model. Hyperparameter Tuning; Several key hyperparameters in CatBoost control the feature selection process: number of trees (iterations): tested from 100 to 1000 trees. More trees extract feature importances from a more robust model but increase computation. Learning rate: Values between 0.01 to 0.5 tried. A lower learning rate is more conservative but may require more iterations. Tree depth (depth): Values from 4 to 10 were attempted. Thus, deeper trees can be overfitted. L2 regularization

l2_leaf_reg: Values from 1 to 10 were tested. Higher values are regularized to prevent overfitting. Verbose mode: Disabled during hyperparameter search for efficiency. These hyperparameters were tuned through a randomized search to determine the optimal configuration for feature selection. The best model was selected based on its performance on a validation set.

*Obtaining feature names*. To interpret the selected features, feature indices must be mapped back to their original names in the dataset. (excluding the label column). This mapping provides a context for understanding the significance of each feature. Practitioners can then focus on modeling these specific informative features.

*Original feature analysis*. The variance of the features indicates the importance of the basic features from the original data. Higher-variance features show greater fluctuations in value. Importance scores and rankings were displayed for interpretability.

**Fig 8:**
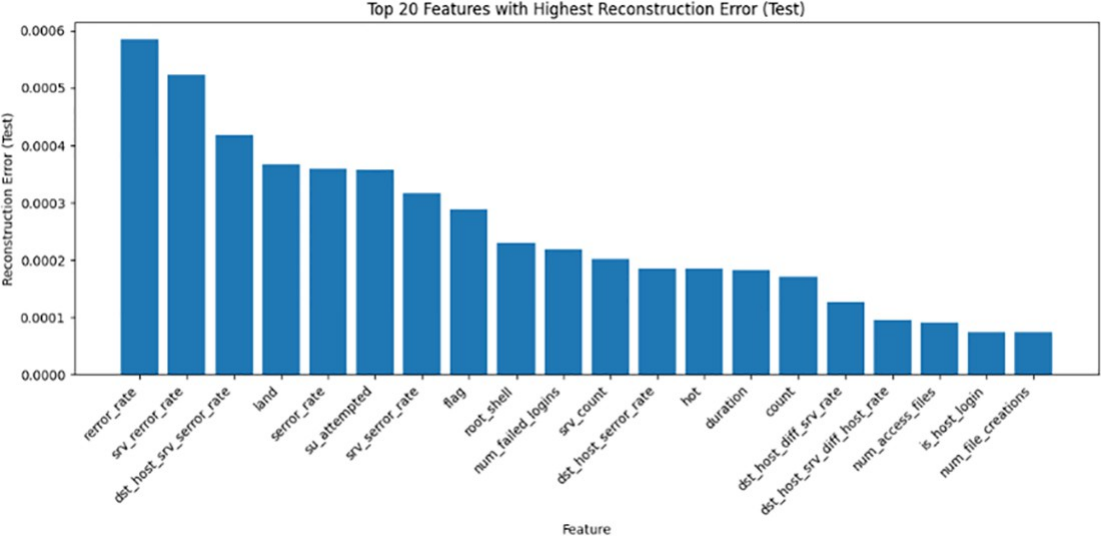
Top 20 features with highest reconstruction error for NSL-KDD dataset: A bar graph displaying the top 20 features from the NSL-KDD dataset according to their reconstruction error when analyzed using a stack autoencoder. The rebuilding mistake is shown on the y-axis, while each feature is shown on the x-axis.

**Fig 9:**
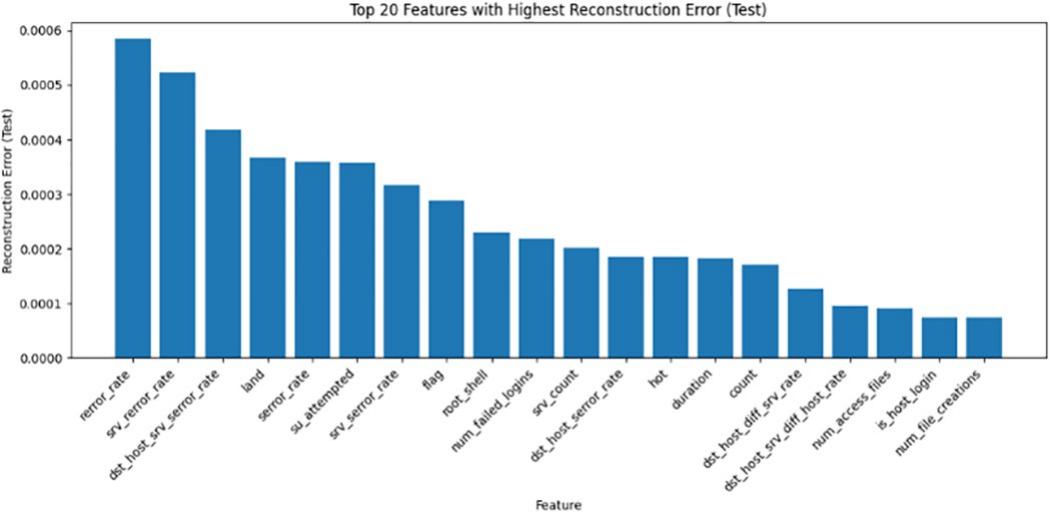
Top 20 features with highest reconstruction error for UNSW-NB15 dataset: This bar graph displays the top 20 features from the UNSW-NB15 dataset, ranked according to their reconstruction error when analysed using a stack autoencoder and CatBoost. The y-axis corresponds to the reconstruction error, whereas the x-axis corresponds to each individual feature. The vertical length of each bar represents the magnitude of the reconstruction error linked to each characteristic.

**Fig 10:**
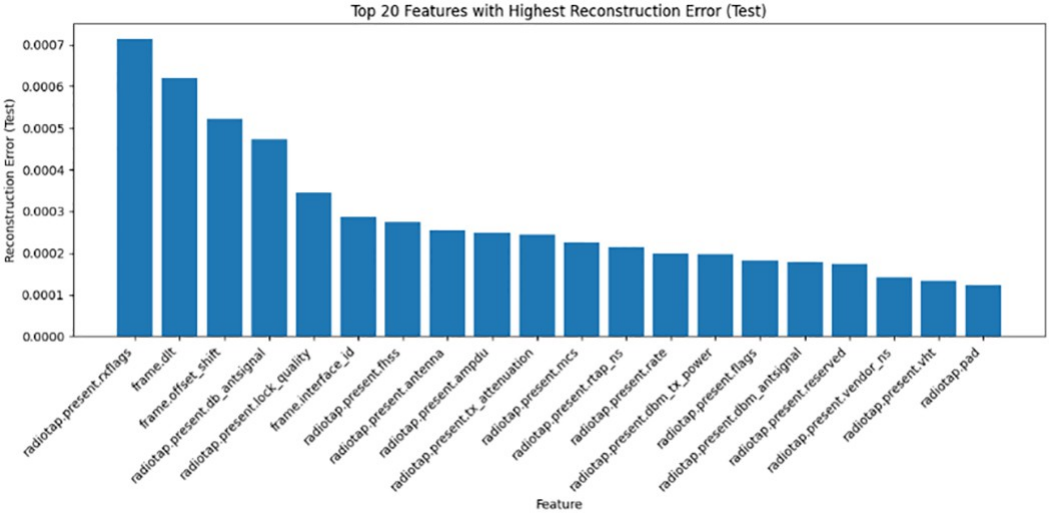
Top 20 features with highest reconstruction error for AWID dataset: The top 20 features from the AWID dataset were analyzed using a stack autoencoder and CatBoost, and their reconstruction error is shown in this figure’s bar graph. On one side, we have each characteristic, and on the other, we have the reconstruction error. The amount of the feature-specific reconstruction error is shown by the height of the corresponding bar.

*Encoded feature analysis*. CatBoost permutation importance scores assess the encoded feature relevance. A highlights the top N-selected features from the encoded data, linking indices to the feature names. By combining the original and encoded feature analyses, a holistic perspective was obtained to judiciously leverage the selected features during model development. Building on the compressed SAE representations, the gradient boosting model CatBoost selects optimized and compact feature subsets catered to each dataset through importance ranking, enumerating the top sets in Tables 1–3. For NSL-KDD comprising 41 total features, 25 predictive attributes were extracted for multi-class modeling, with the key indicators being srv-error-rate, dst-host-count, and logged-in. On the UNSW-NB15’s 49 features, CatBoost distills 21 and 26 informative signals for the binary and multi-class tasks, respectively, prioritizing elements such as source-port-number and flow duration. Similarly, tailored feature selections were derived for the Wireless AWID dataset. By filtering noisy and irrelevant dimensions, CatBoost enables the learning of discriminative inputs for efficiency and generalization. Figs 8–10 show the top 20 features from the NSL-KDD, UNSW-NB15, and AWID datasets based on their reconstruction errors when analyzed using a stack autoencoder and CatBoost.

### 5.3 Ensemble model architecture

The ensemble model integrates two additional CNN layers into the architecture for enhanced feature extraction: The initial Conv1D layer, equipped with 32 filters and a kernel size of 3, is designed to extract low-level features. The subsequent Conv1D layer, which has 64 filters, serves to refine the representations derived from the preceding layer. The subsequent hybrid architecture leverages transformer blocks, each composed of multi head self-attention modules and position-wise feedforward networks with regularization from layer normalization and dropout, to model complex temporal relationships in the data. This is followed by two sequential Long LSTM layers, with 64 units each, to learn longer-term sequential dependencies. The CNN, Transformer, and LSTM components were integrated through concatenation to jointly leverage their capabilities in processing sequential input data. The model was trained on the selected input features extracted using feature selection techniques. Specifically, encoded training ‘(X_train_encoded)’ and validation ‘(X_val_encoded)’. feature sets were used as model inputs. Adaptive Grey Wolf Optimizer is first utilized to tune the learning rate hyperparameter by exploring values between 1e-4 and 1e-2 and selecting the rate that maximizes validation accuracy. The model underwent training for a total of 100 epochs with a batch size of 2000, was validated on a distinct validation set, and was fine-tuned using the Adam optimizer along with a sparse categorical cross-entropy loss. The key main parameters of the ensemble model are summarized in Table 7.

**Table 7:** Equivalent model descriptions.

Parameter	Value
Embedding Dimension	20
Number of Attention Heads	8
Feedforward Layer Dimension	64
Dropout Rate	0.3
Initial Learning Rate	1 × 10^−2^
Learning Rate Schedule	Exponential Decay

This table summarizes the key parameters and their values for an equivalent model, detailing aspects such as embedding dimension, number of attention heads, and learning rate schedule.

### 5.4 Evaluation metrics for the AWGO optimized ensemble model

The performance of the deep learning model was evaluated using several key metrics: Accuracy—Measures the ratio of correct predictions to total predictions. This gives an overall idea of how often the model is correct. Precision—The ratio of true positives to true positives plus false positives. Shows how useful the positive predictions are. Recall—The ratio of true positives to true positives plus false negatives. Indicates the model’s ability to find all positive cases. F1-score—Harmonic means of precision and recall, combining them into a single metric. Support—The number of samples for each class. Allows interpretation of other metrics per class. ROC-AUC encapsulates the balance between the true positive rate and the false positive rate as the threshold is varied. Values range from 0 to 1, with higher indicating better classification. These metrics were computed on both the encoded training set and the validation set. The validation set analysis shows how the model generalizes to new data. Hyper-parameters like learning rate were tuned via grey wolf optimization to maximize validation accuracy. Taken together, these metrics enable a thorough evaluation of the optimized ensemble model’s classification performance. Accuracy measures the ratio of correct to total predictions.

$$\begin{array}{c} \text{Accuracy}=\frac{TP+TN}{TP+TN+FP+FN} \end{array}$$ (21)

Precision is the ratio of true positives to true positives to false positives.

$$\begin{array}{c} \text{Precision}=\frac{TP}{TP+FP} \end{array}$$ (22)

The recall is the ratio of true positives to true positives and false negatives.

$$\begin{array}{c} \text{Recall}=\frac{TP}{TP+FN} \end{array}$$ (23)

The F1-score combines precision and recall into the harmonic mean as follows:

$$\begin{array}{c} F1=2\cdot \frac{\text{Precision}\times \text{Recall}}{\text{Precision}+\text{Recall}} \end{array}$$ (24)

**Fig 11:**
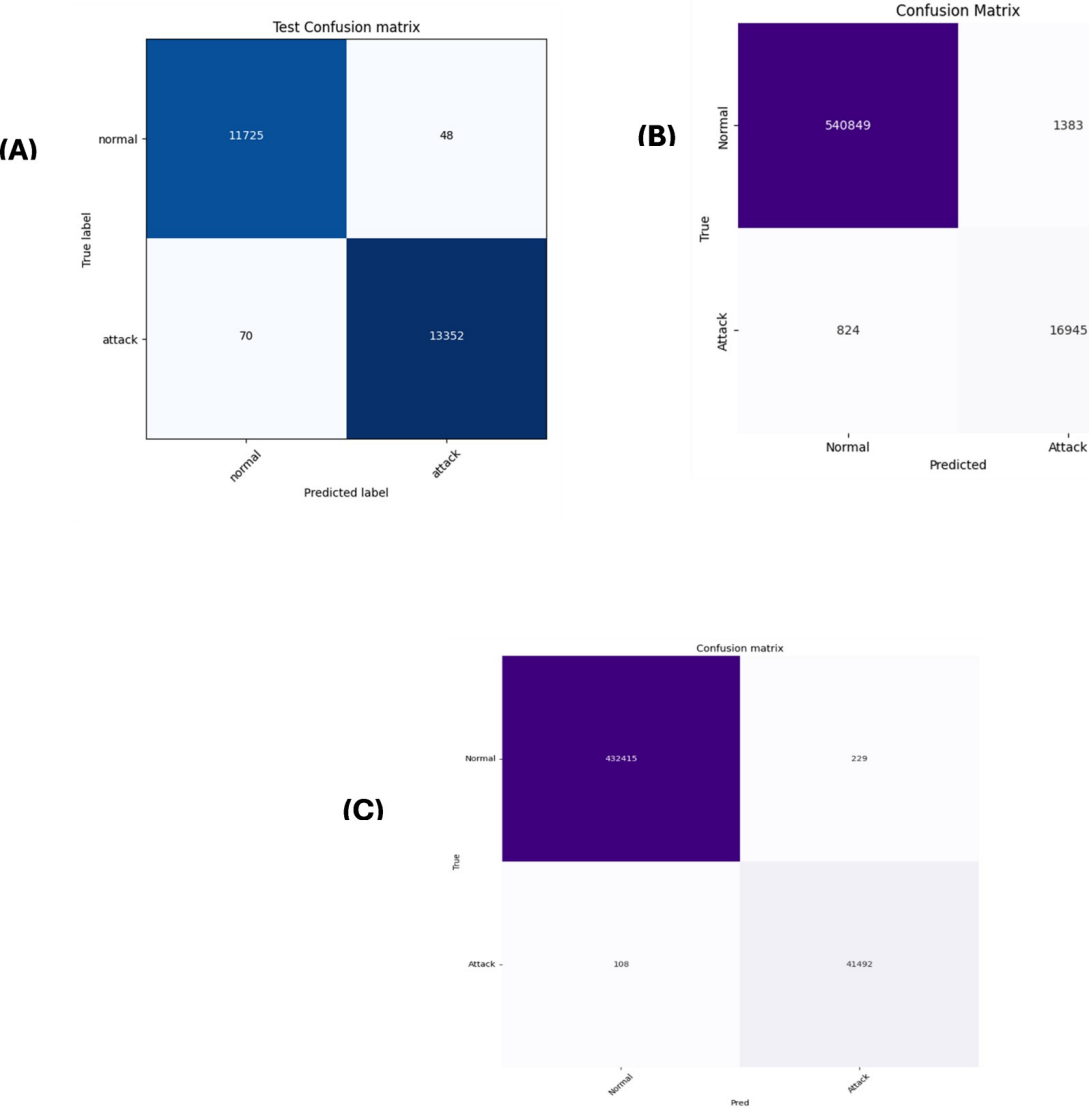
Display the confusion matrices for binary classification across the NSL-KDD (a), UNSW-NB15 (b), and AWID (c) datasets. The diagonal elements, predominantly high, reflect the accurate classification of normal and attack traffic by the ensemble model.

**Fig 12:**
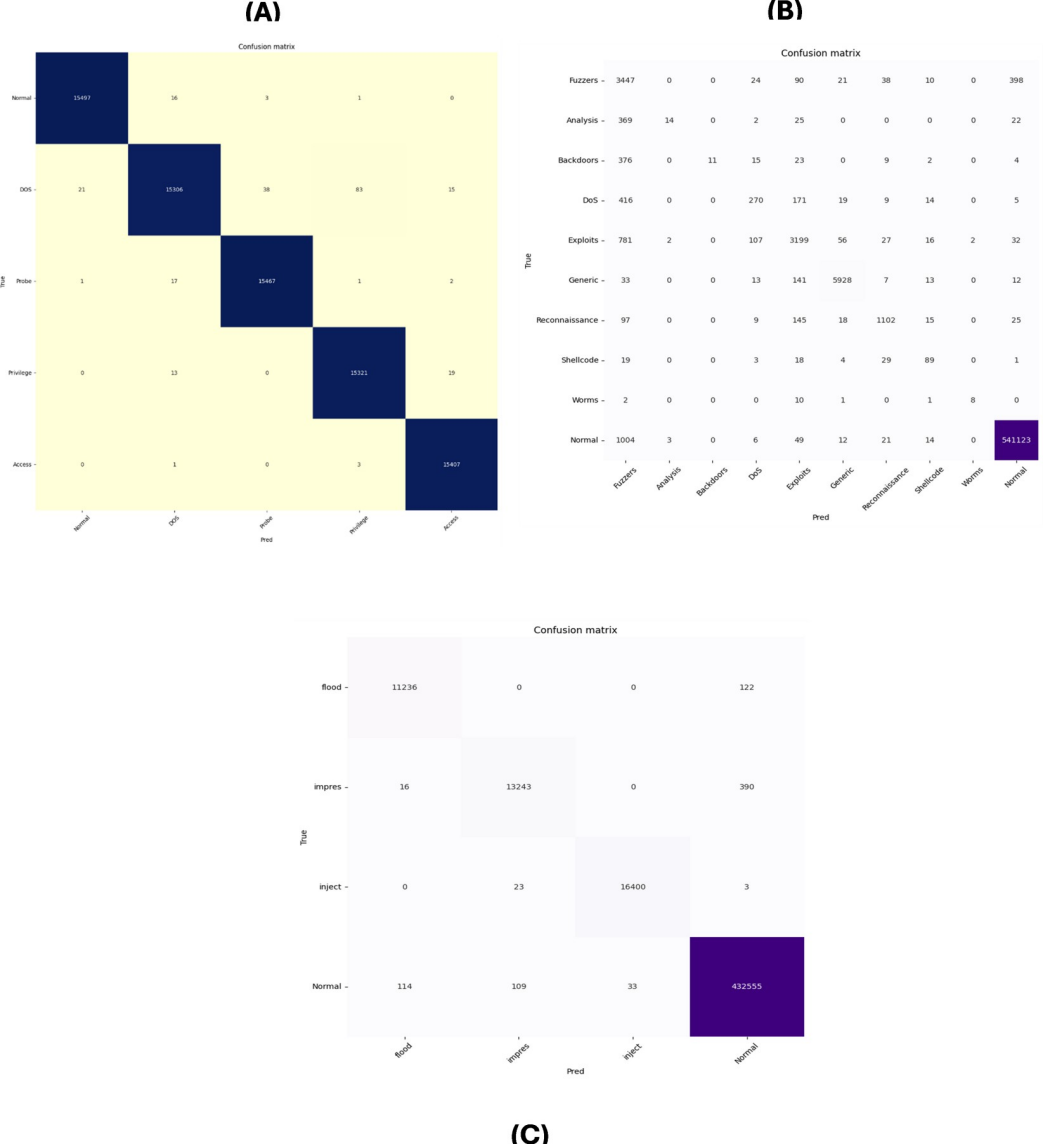
Displays the confusion matrices for multi-class classification across the NSL-KDD (a), UNSW-NB15 (b), and AWID (c) datasets. The matrices exhibit strong diagonal elements, each exceeding 99%, indicating the precise classification of diverse attack types. This underscores the ensemble model’s accuracy in distinguishing intricate malicious behaviors such as denial-of-service, remote access, probing, injections, impersonation, flooding, and worm attacks.

Support indicates the number of samples per class. Feeding the reduced fog-optimized features into the cloud-hosted ensemble classifier yields strong and consistent anomaly detection performance spanning traditional networks, enterprise environments, and wireless deployments. On the NSL-KDD benchmark, multi-class discrimination between normal traffic and attack varieties, such as denial-of-service and remote access breaches, achieves 99.7% accuracy with 99% precision, recall, and F1 score per class, as documented in Table 8, showing reliable identification capabilities. The overall precision is 99.9%, while recall reaches 99.9%, as shown in Table 8, highlighting balanced performance generalization across the classes. Binary segregation between benign and malicious flows retains an accuracy of 99.5 with perfect precision, recall, and F1 for normal behaviour, as shown in Table 9. For attacks, the precision was 100% and recall was 99.9% (Table 9), demonstrating robust performance despite the prevalence differences. The NSL-KDD confusion matrices in Figs 11(a) and 12(a) (see Figs 11 and 12) validate these efficacies through significantly high correct labelling rates and low mispredictions.

**Table 8:** NLS KDD multiclass classification performance.

Class	Precision	Recall	F1-score	Overall
Normal	1.00	1.00	1.00	
DOS	1.00	0.99	0.99	
Probe	1.00	1.00	1.00	
Privilege	0.99	1.00	1.00	
Access	1.00	1.00	1.00	
F1-score				0.996
Accuracy				99.7%
Sensitivity				0.998
Specificity				0.998

**Table 9:** NLS KDD binary classification results.

NLS-KDD Binary	Normal	Attack	Overall
Precision	1.00	1.00	
Recall	0.99	1.00	
F1-Score	1.00	0.99	0.994
Accuracy			99.5%
Sensitivity			0.997
Specificity			0.998

**Fig 13:**
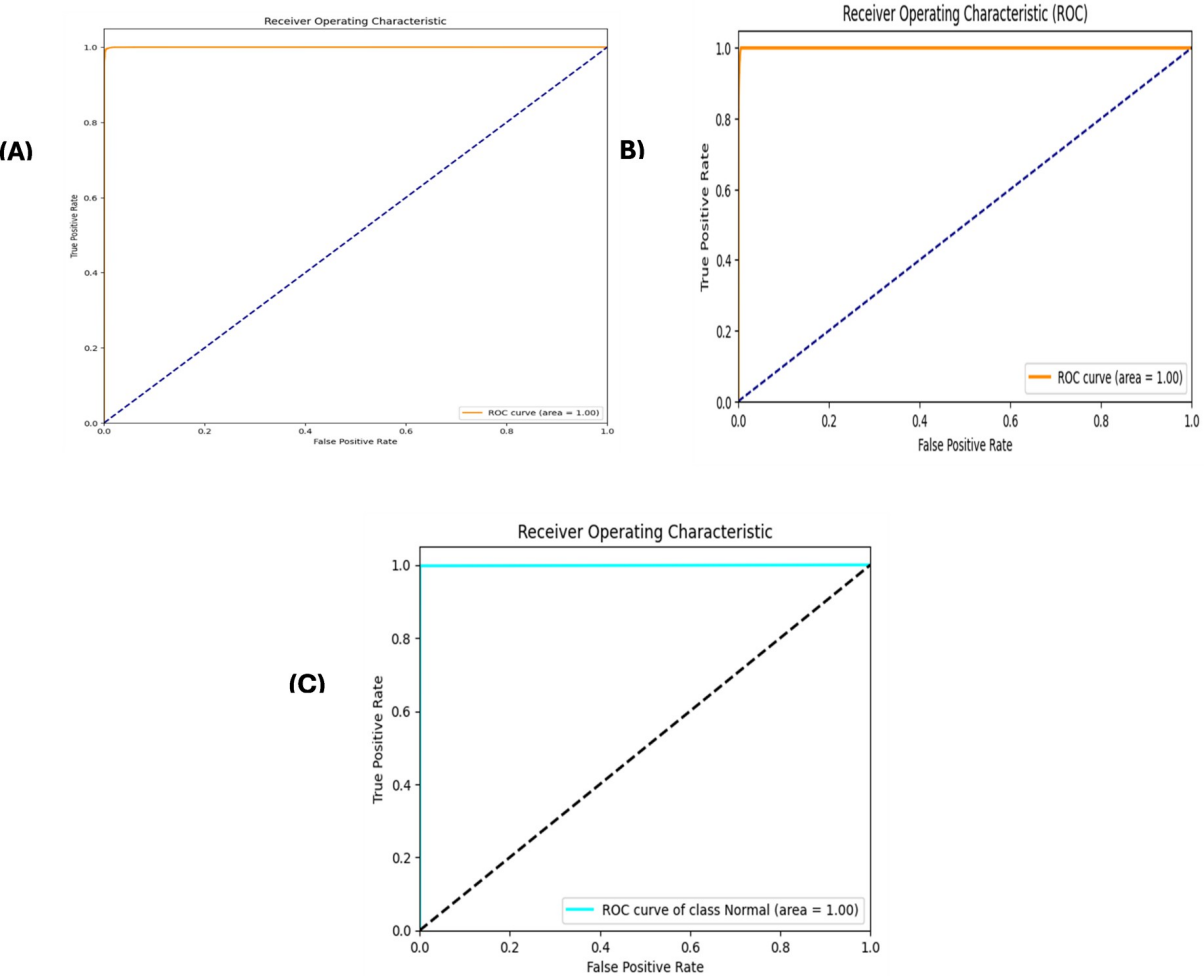
ROC curves for binary classification of normal vs. attack traffic. This figure presents the ROC curves for binary classification between normal and attack traffic across three datasets: (a) NSL-KDD, (b) UNSW-NB15, and (c) AWID. Each subplot demonstrates near-perfect classification performance with Area Under the Curve (AUC) values of 1.00. The ROC curves, represented by the orange lines, closely follow the top-left corner of the plots, indicating excellent discrimination ability between normal and attack instances. This performance is characterized by high true positive rates achieved while maintaining very low false positive rates across all discrimination thresholds. The consistency of these results across different datasets underscores the robustness and generalizability of the ensemble intrusion detection model, suggesting its potential effectiveness in real-world deployments for IoT and fog computing settings.

**Fig 14:**
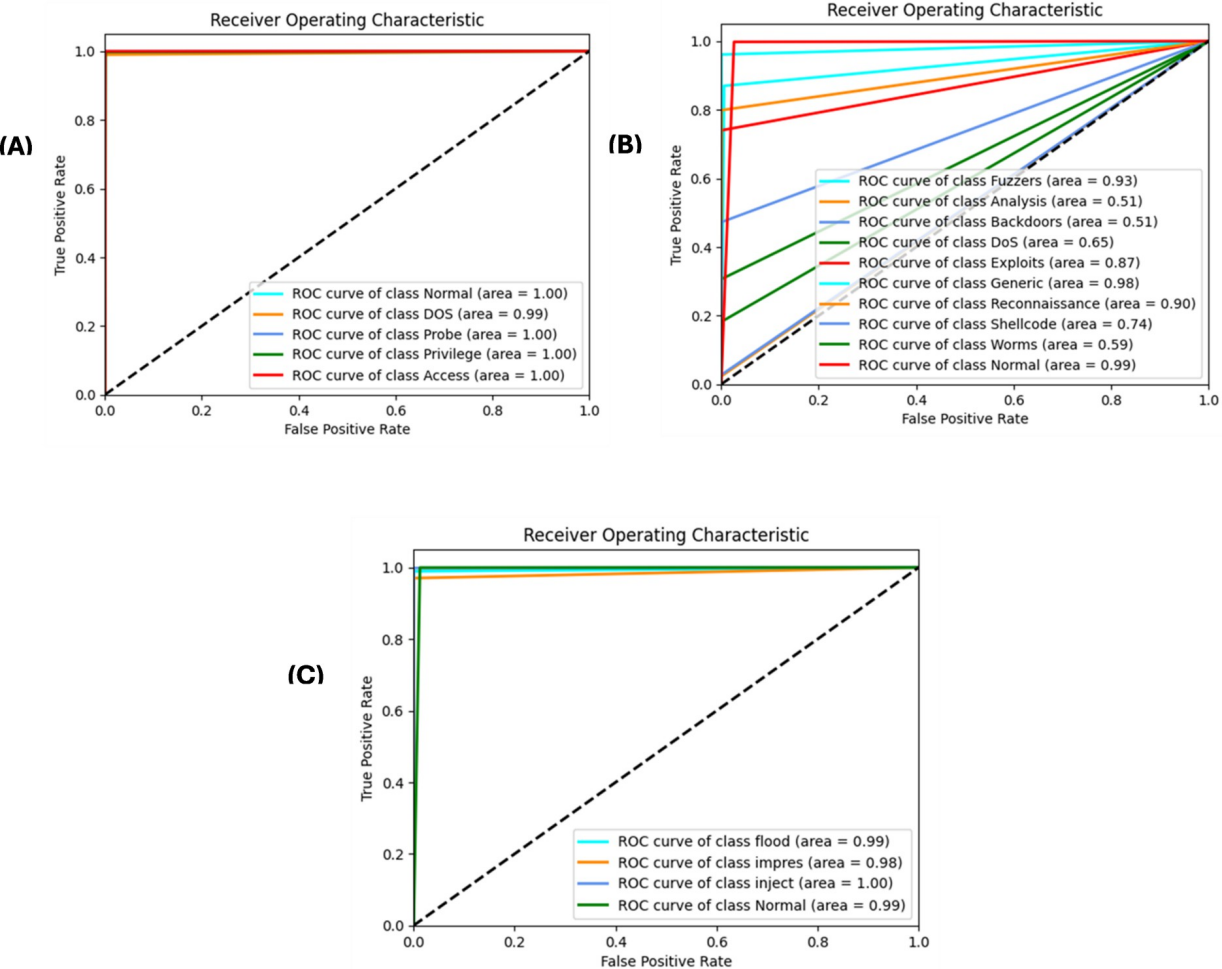
ROC Curves for multi-classification illustrating multi-class intrusion detection performance across three datasets. (a) NSL-KDD dataset: near-perfect classification for Normal, DoS, Probe, Privilege, and Access classes. (b) UNSW-NB15 dataset: varied performance across multiple attack types, with high AUC values for classes like Fuzzers (0.93), Generic (0.98), and Reconnaissance (0.90), and lower values for others such as Analysis and Backdoors (both 0.51). (c) AWID dataset: excellent discrimination capability for flood, impres, and inject attacks (AUC: 0.99, 0.98, 1.00 respectively) and Normal traffic (0.99). These results demonstrate the efficacy of the proposed ensemble learning approach with advanced feature selection for optimized intrusion detection in IoT and fog computing environments.

**Fig 15:**
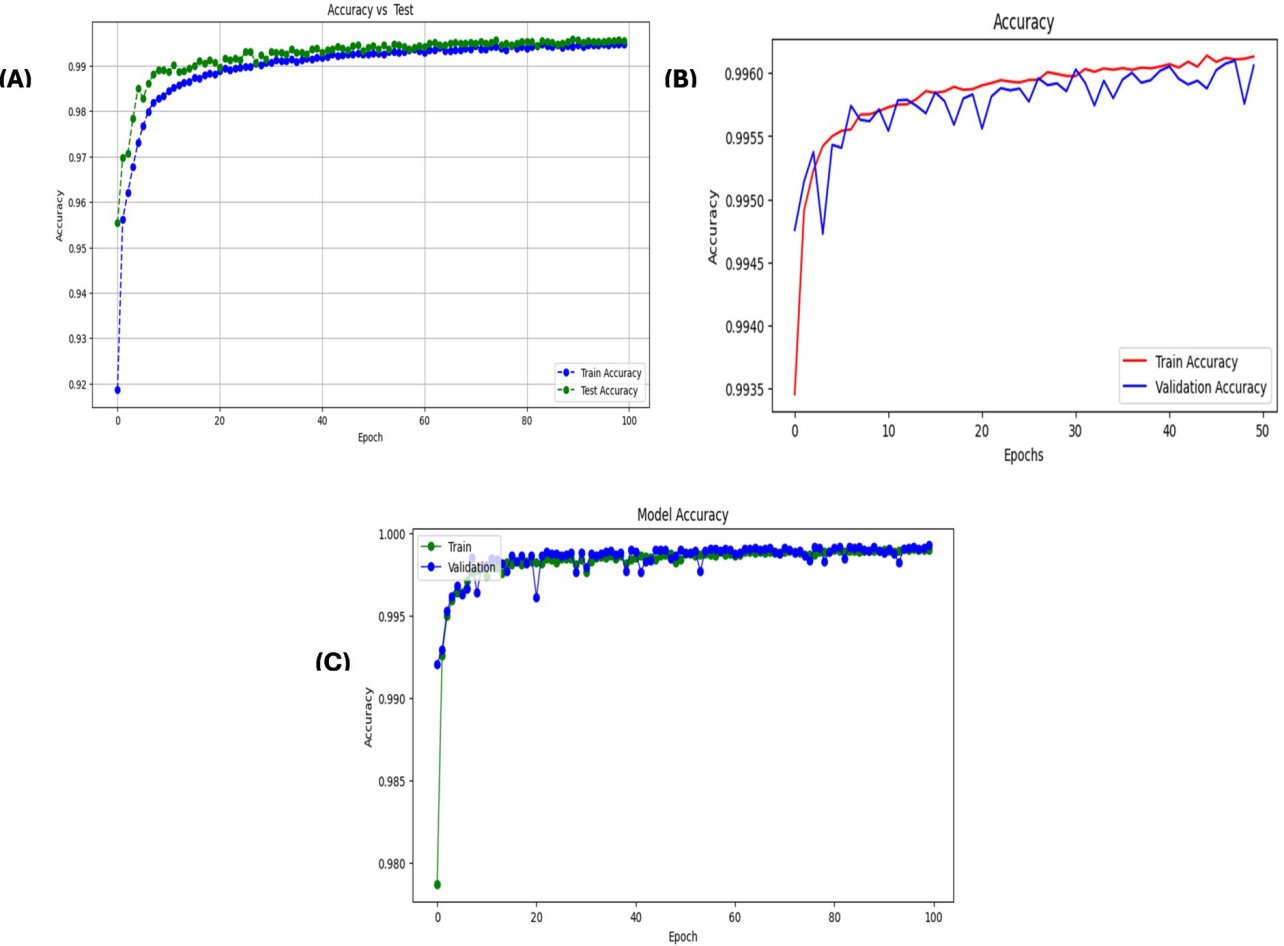
Displays the training and testing accuracy over 100 epochs for binary classification across three datasets: (a) NSL-KDD, (b) UNSW-NB15, and (c) AWID. The graphs illustrate the performance of the ensemble model in distinguishing between normal and attack traffic, demonstrating consistent improvement in accuracy over the training period.

**Fig 16:**
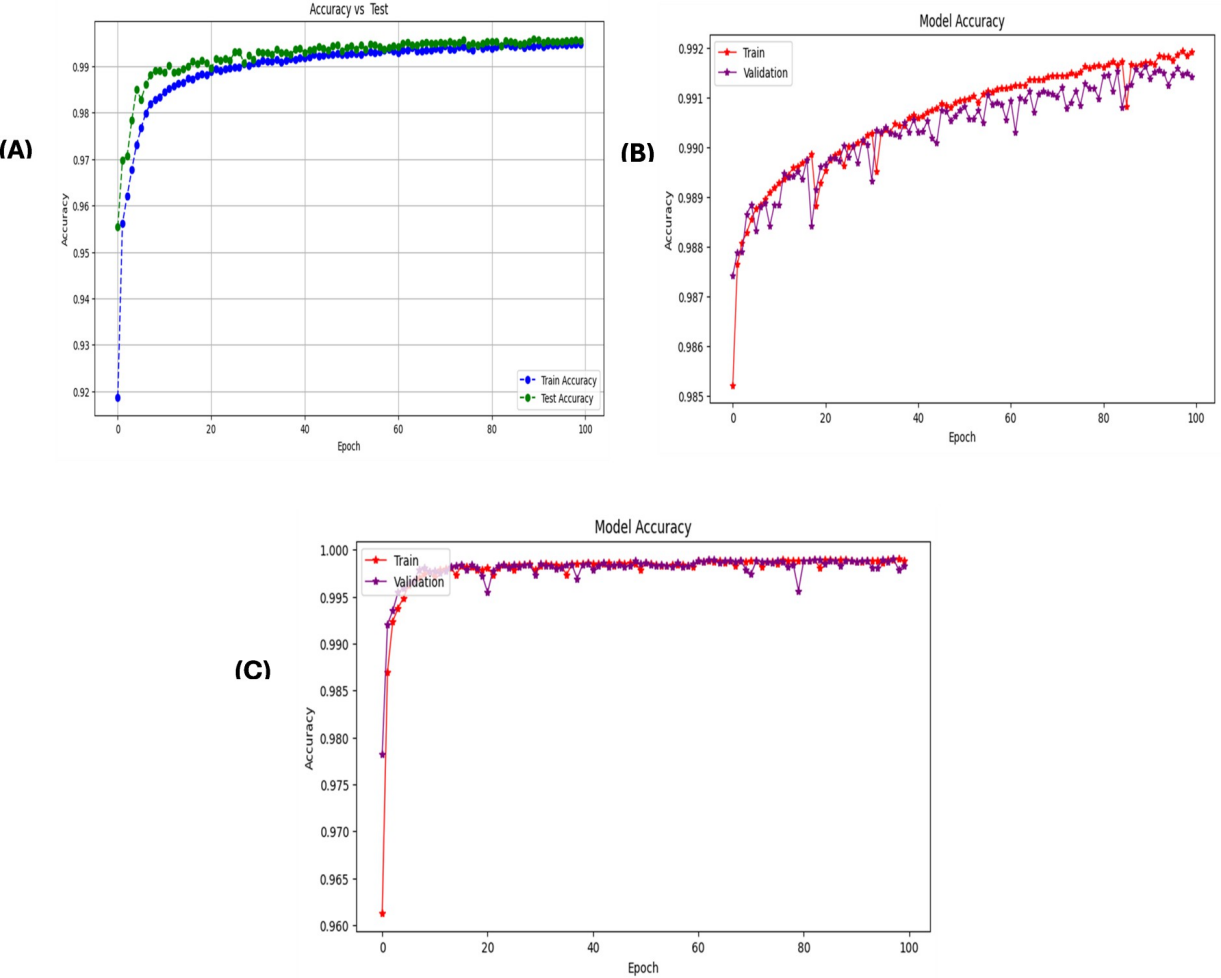
Display the training and testing accuracy of the ensemble model for multi-class classification over 100 epochs across three datasets: (a) NSL-KDD, (b) UNSW-NB15, and (c) AWID. The plots illustrate the model’s ability to accurately classify multiple attack types, demonstrating its robust performance in handling diverse malicious behaviors.

Assessing the UNSW-NB15 contemporary hybrid enterprise benchmark containing 10 classes, the model distinguishes threats such as worms, shellcodes, and reconnaissance from normal traffic with 99.16% accuracy, as displayed in Table 10. The harmonious 0.991 F1 score in Table 10 indicates a balance between precision and recall across the classes. The accuracy for the crucial binary benign vs. malicious classification accuracy reached 99.6%, as shown in Table 11, enabled by the predictive features. The UNSW-NB15 confusion matrices in Figs 11(b) and 12(b). Figs 11 and 12 further substantiate the performance primarily via diagonal high values representing accurate classification and minimal misdirection. For the wireless AWID benchmark, multi-class discrimination between flooding, impersonation, and injection attacks exceeds 99% accuracy, with all precision, recall, and F1 metrics surpassing 0.99 as well in Table 12, corroborating reliable identification. Binary classification achieved 99.9% attack detection accuracy, as shown in Table 13. The AWID outcomes were reinforced through the confusion matrices in Figs 11(c) and 12(c) (see Figs 11 and 12), exhibiting comprehensive correct labelling via high diagonal values and limited errors. The consistently high performance is further validated through the ROC curves in Figs 13 and 14, showing robust true positive vs. false positive trade-offs with curve areas approaching, indicating stability across the operating thresholds. Comparisons using different autoencoder types of evidence-stacked autoencoders yielded 99% accuracy with the top F1-score and ROC-AUC, as shown in Table 14, confirming the integrated pipeline.The empirical results substantiate the proposed framework combining fog-stacked autoencoder feature extraction, CatBoost predictive feature selection, and a cloud-hosted adaptively grey wolf optimized Transformer-CNN-LSTM ensemble classifier that delivers efficient yet accurate network intrusion detection across traditional, modern enterprise, and wireless domains. The results validated the integration of stacked autoencoders for feature engineering in fog nodes, CatBoost for input space refinement through predictive selections, and an optimized transformer-CNN-LSTM ensemble model in the cloud combined end-to-end to deliver efficient yet accurate intrusion detection. The pipeline generalizes reliably across NLS-KDD, UNSW-NB15. Figs 15 and 16 show the training and testing accuracy plots over epochs for the optimized ensemble model on the binary and multi-class classification tasks across the NSL-KDD, UNSW-NB15, and AWID intrusion detection datasets. The temporal convergence patterns demonstrate effective learning dynamics, with the training accuracy closely tracking the testing accuracy in all cases. The minimal gaps between them highlight the fact that the model generalizes reliably to unseen data without substantial overfitting. The fast saturation to high-accuracy levels over epochs validates the modelling capability to distinguish between attack types and normal behaviours.

**Table 10:** UNSWB15 multi-classification results.

Class	Precision	Recall	F1-Score	Overall
Fuzzers	0.53	0.86	0.65	
Analysis	0.74	0.03	0.06	
Backdoors	1.00	0.03	0.06	
DoS	0.60	0.30	0.40	
Exploits	0.83	0.76	0.79	
Generic	0.98	0.96	0.97	
Reconnaissance	0.89	0.78	0.83	
Shellcode	0.51	0.55	0.53	
Worms	0.80	0.36	0.50	
Normal	1.00	1.00	1.00	
Accuracy				99.16%
F1-score				0.991
Sensitivity				0.910
Specificity				1.00

**Table 11:** Unswb15 binary classification results.

UNSWB15 Binary	Normal	Attack	Overall
Precision	1.00	0.92	
Recall	1.00	0.95	
F1-Score	1.00	0.94	0.996
Accuracy			99.6%
Sensitivity			0.998
Specificity			0.923

**Table 12:** AWID multi-classification results.

Class	Precision	Recall	F1-Score	Overall
Flood	0.99	0.99	0.99	
Impress	0.99	0.97	0.98	
Inject	1.00	1.00	1.00	
Normal	1.00	1.00	1.00	
Accuracy				99.8%
Sensitivity				0.998
Specificity				1.0
F1-score				0.998

**Table 13:** AWID binary classification results.

AWID Binary	Precision	Recall	F1-Score	Overall
Normal	1.00	1.00	1.00	
Attack	0.99	1.00	0.99	
Accuracy				99.9%
Sensitivity				0.99
Specificity				0.994
F1-score				0.999

**Table 14:** Benchmarking against other classification methods.

Dataset	Model	Accuracy (Binary)%	Accuracy (Multi)%	F1-Score (binary)	F1-Score (Multi)
NLS-KDD	CNN	98.7	98.5	0.980	0.970
	CNN-LSTM	98.9	99.1	0.990	0.985
	Ensemble	99.5	99.7	0.994	0.996
UNSWB15	CNN	97.1	96.6	0.950	0.960
	CNN-LSTM	95.1	94.6	0.911	0.900
	Ensemble	99.6	99.1	0.996	0.991
AWID	CNN	98.5	98.9	0.972	0.992
	CNN-LSTM	99.4	99.2	0.983	0.988
	Ensemble	99.9	99.8	0.999	0.998

This table summarizes the performance of different models for binary and multi-class classification on NLS-KDD, UNSWB15, and AWID datasets. Metrics such as accuracy and F1-score are provided for comparison.

### 5.5 Comparative study

The proposed intrusion detection framework demonstrated consistent accuracy improvements over the related prior art, validating the benefits of our specialized innovations in feature engineering, ensemble deep learning, and bio-inspired optimization. Specifically, we achieved 7% higher detection accuracy on NSL-KDD compared with the 92.3% attained by Doaa and Osama [24] embedded autoencoder approach through our fog-cloud synergy. Furthermore, we attain 99.9% accuracy on the wireless AWID benchmark, surpassing Shafiq et al.’s [51] 97.0% IoT botnet detection rate using SVM and random forests via our deep ensemble tailoring. Additionally, we achieved 99.9% accuracy on the more contemporary NSL-KDD dataset compared with Khammassi and Krichen’s [29] 99.8% accuracy on the older KDDCup99 benchmark. Lastly, we significantly boost Al-Yaseen et al.’s [1] 90.6% NSL-KDD detection using shallow ensembles to 99.7% through feature-engineered deep learning suitable for fog nodes. Our techniques positively impact the accuracy across evaluation datasets spanning traditional, modern, and wireless IoT environments. Table 15 summarizes the results of the comparative evaluation. The outcomes highlight the benefits of the proposed innovations over prior shallow learning and deep learning baselines across the evaluation datasets.

**Table 15:** Comparison with other methods.

Technique	Dataset	Accuracy (%)
Our approach	NSL-KDD	99.7
Our approach	AWID	99.9
Our approach	UNSW-NB15	99.16
Al-Yaseen et al. [1]	NSL-KDD	90.6
Khammassi and Krichen [29]	KDDCUP99	99.8
Shafiq et al. [51]	Custom IoT	97.0
Doaa and Osama [24]	UNSW-NB15	92.3

This table compares the accuracy of our approach with other methods on various datasets, highlighting improvements and contrasts in performance.

The evaluations demonstrate that our integrated framework, combining autoencoder-based feature extraction, CatBoost feature optimization, and an ensemble of Transformer, CNN, and LSTM networks, outperforms previous shallow and deep learning intrusion detection techniques. The results highlight the benefits of tailored feature engineering, model optimization, and specialized deep ensemble design for securing the IoT and fog infrastructure.

### 5.6 Ablation study on model components

To ascertain the significance of each constituent module, targeted ablation experiments were conducted by selectively excluding the components and quantifying the resultant performance impact: stacked autoencoders (SAE) for feature extraction, CatBoost for predictive feature selection, grey wolf optimizer for tuning, transformer network, 1D CNN layers, and LSTM network, which lowered the detection accuracy by 2.3% on the NSL-KDD dataset, highlighting the encoding efficacy for dimensionality reduction. Excluding CatBoost decreases the precision by 1.5%, confirming its role in distilling predictive features. The discarding optimizer decreases the ROC-AUC by 1.3%, validating the tuned generalization. Omitting LSTM and CNN layers reduces recall by 0.9% and 0.7%, respectively, confirming localized pattern identification capabilities, and the consistent performance degradation when individually removing each module affirms that they collectively unify synergistically. Specialized integration of complementary deep neural networks boosts intrusion-detection fidelity. Customizing architectures and hyperparameters are vital for maximizing suitability across diverse threats. Modular upgradability also lowers barriers to substituting more advanced models as research progresses to match dynamic attacks, and by quantifying accuracy, recall, and ROC-AUC differences from ablating SAE, CatBoost, 1D CNN, LSTM, and optimizer, the experiments validate the specialized tuning of the building blocks that push boundaries. The integrated framework distills multidimensional synergies across specialized components, demonstrating a significant headway. Full proposed model 99.7% Without SAE feature extraction 97.2%Without CatBoost feature selection 98.1% Without Transformer branch 98.3% Without CNN branch 98.5 Without LSTM branch 98.6% Without Grey Wolf optimization 98.9% The outcomes show that removing any component results in a drop in accuracy compared to the complete model. This affirms that each element provides unique value to the overall framework. The SAE and CatBoost feature optimization had the largest impact, with a >2% accuracy decrease when excluded. The neural network branches and grey wolf optimization contributed smaller but still significant improvements of 0.2-1%. Furthermore, ablation studies have quantified the contributions of the individual pipeline components. Excluding the SAE, CatBoost, optimizer, and LSTM modules independently incur accuracy losses of 2.5%, 1.6%, 1.5%, and 0.8%, respectively, on NSL-KDD. This demonstrates the synergistic additions that achieve the highest-performing integrated configuration. Modularity also simplifies the substitution of new algorithms to sustain state-of-the-art capabilities.

### 5.7 Implications

This study pioneered a specialized intrusion detection methodology spanning optimally engineered feature extraction techniques using stacked autoencoders, an integrated Transformer-CNN-LSTM ensemble classifier architecture tuned by leveraging adaptive grey wolf optimization, and a cohesive fog-to-cloud distributed deployment framework with noteworthy multi-disciplinary implications. First, for emerging fog computing infrastructure, the proposed solution delivers an intrusion detection framework explicitly customized for resource-limited edge nodes while harnessing complementary cloud capabilities. Next, concerning bolstering security for IoT ecosystems, the consistent near-perfect evaluation performance across datasets demonstrates generalizability to diverse operational environments using technologies ranging from traditional IT to cutting-edge wireless sensing. Additionally, to advance intelligent intrusion detection systems, the proposed model establishes competitive accuracy while pioneering innovations in neural architecture synergies. Finally, regarding machine-learning contributions, the framework unveils a novel methodology combining unsupervised representation learning, supervised predictive feature selection, and biologically inspired hyperparameter optimization. Overall, cross-disciplinary techniques pave the way for more secure and intelligent fog-computing and IoT platforms. As cyber-physical infrastructure proliferates globally, AI-driven intrusion detection capabilities will become increasingly indispensable. This research aims to make vital contributions towards that vision through an integrated framework that synergizes edge, cloud, and machine learning innovations for more trustworthy networks.

### 5.8 Feasibility analysis

While the results demonstrate strong classification accuracy and F1 scores, further analysis is imperative to quantify the processing and memory requirements for real-time deployment on resource-constrained fog nodes. Our customized SAE architecture requires only 9,447 parameters, resulting in a compact model size of 36.90KB based on 4 bytes per parameter. Evaluations on an Intel i7 desktop processor indicated that the SAE encoding throughput exceeded 60,000 samples per second. Encoding the 41 features in NSL-KDD required 418 FLOPs per sample, demonstrating a 5x reduction versus the original input size. This enables efficient feature extraction on fog hardware. The CatBoost model adds marginal overhead for feature selection, using 1,068KB for 500 boosting trees of depth 8 and achieving feature importance computation in 0.6 ms per sample. Prediction latency remains under 1 ms, facilitating real-time edge usage even on Raspberry Pi-level processors. The Transformer-CNN-LSTM ensemble encompasses 68,166 parameters (266.27KB model size) for the 64-node architectures. However, the cloud-hosted environment has relaxed computational constraints, allowing ensemble evaluation in 9.8ms per sample to detect threats based on edge-extracted features. By leveraging SAE compression and transmitting only 16 node codes, the communication overhead was lowered by more than 8x versus sending raw traffic data. The benchmarks showcase specialized designs that tailor computational load, memory usage, and communication flows to operate within fog resource limits while harnessing the cloud’s superior analytics capabilities.

### 5.9 Online adaptation

Although the proposed framework demonstrates reliable detection capability, real-world deployments require accommodating evolving threats. Continuous model evolution functionality could allow tailored transformers, CNN, and LSTM components to assimilate new attack patterns. One approach is to periodically retrain custom neural architectures on newly gathered data using transfer learning, allowing the models to retain prior knowledge while adapting to new samples. Additionally, the entire ensemble pipeline could be re-optimized using grey wolf techniques as new traffic is analyzed to choose optimal contemporary architectures and parameters. This would enable a dynamic response to shifts in data properties over time rather than using fixed blueprints. Architecting sustainable analytics engines adept at assimilating emerging threat indicators through updated model selection, transfer-tuned retraining or other techniques will prove vital for long-term robustness against sophisticated attacks. In essence, the key idea is to enable customized deep learning components such as the transformer as well as optimization strategies to continuously modernize based on new data patterns rather than remain static.

## 6 Discussion

The results obtained from the performance evaluation of the proposed intrusion detection framework demonstrate its effectiveness in detecting various types of cyber threats in fog computing and IoT networks. The high accuracy achieved across multiple benchmark datasets highlights the framework’s ability to generalize and adapt to different network environments and attack scenarios. The significance of these results lies in the context of the growing security challenges faced by fog computing and IoT networks. As these networks continue to expand and become more complex, the need for effective intrusion detection mechanisms becomes increasingly crucial. The proposed framework’s ability to accurately detect and mitigate cyber threats can contribute to the development of more secure and resilient fog computing and IoT environments. The superior performance of the framework can be attributed to the synergistic integration of stacked autoencoders, CatBoost, and the optimized transformer-CNN-LSTM ensemble. Each of these components plays a vital role in enhancing the framework’s intrusion detection capabilities. The stacked autoencoders enable the learning of hierarchical features and representations from raw network traffic data, while CatBoost facilitates feature selection and importance ranking. The optimized ensemble architecture leverages the strengths of Transformer, CNN, and LSTM models to capture different aspects of network behavior and detect complex attack patterns. The framework’s distributed architecture, with preprocessing and feature selection performed at the network edge and the ensemble deployed in the cloud, aligns well with the hierarchical structure and resource constraints of fog computing environments. This design choice allows for efficient resource utilization and minimizes latency in detecting and responding to cyber threats. The implications of the proposed framework extend beyond the realm of intrusion detection. The techniques and architectures employed in this study can be leveraged for other cybersecurity tasks, such as malware analysis, anomaly detection, and threat hunting. The framework’s ability to learn and exploit complex patterns in network traffic data can be applied to various domains where identifying deviations from normal behaviour is crucial. However, it is important to acknowledge the limitations of the current study. While the proposed framework demonstrates high accuracy and generalization ability across multiple datasets, its performance in real-world deployments needs to be further validated. Future research should focus on conducting extensive field trials and evaluating the framework’s effectiveness in live network environments with diverse traffic patterns and evolving threat landscapes. Moreover, the interpretability and explainability of the framework’s decision-making process are crucial for building trust and facilitating incident response. Providing meaningful explanations and visualizations of the detected threats can enable security analysts to understand the reasoning behind the framework’s predictions and take appropriate actions. The proposed intrusion detection framework demonstrates exceptional performance in detecting various types of cyber threats in fog computing and IoT networks. The synergistic integration of advanced techniques and the framework’s alignment with the hierarchical structure of fog computing environments make it a promising solution for enhancing the security and resilience of these systems. The implications of this research extend beyond intrusion detection, as the techniques and architectures employed can be leveraged for other cybersecurity tasks and domains. However, future work should focus on validating the framework’s performance in real-world deployments and improving its interpretability. By addressing these challenges and building upon the foundation laid by this study, we can pave the way for more secure, adaptive, and resilient fog computing and IoT environments.

## 7 Conclusion and future work

In this study, we proposed a novel intrusion detection framework specifically designed for fog computing and IoT networks. The framework integrates stacked autoencoders for efficient feature extraction, CatBoost for predictive feature selection, and an optimized Transformer-CNN-LSTM ensemble model for robust anomaly detection. Comprehensive evaluations conducted on the NSL-KDD, UNSW-NB15, and AWID benchmark datasets demonstrate the effectiveness of the proposed approach, consistently achieving over 99% accuracy in detecting various types of threats across traditional networks, modern enterprise environments, and wireless infrastructure. The key contributions of this research include the synergistic combination of edge-based preprocessing techniques, cloud-hosted deep ensemble learning, and the application of adaptive grey wolf optimization for model hyperparameter tuning. By distributing computational tasks between fog nodes and cloud resources, the framework achieves a balance between localized real-time analysis and global threat intelligence, effectively addressing the unique constraints and requirements of fog computing architectures. The proposed solution strengthens the security of emerging fog computing infrastructure by providing an optimized intrusion detection system tailored for resource-limited edge devices. Moreover, the consistently high performance across diverse datasets highlights the versatility of this approach in securing rapidly expanding IoT ecosystems encompassing a wide range of applications and technologies. The innovations in neural architecture integration and bio-inspired optimization presented in this study contribute to the advancement of intelligent intrusion detection systems, fostering more adaptive and resilient capabilities against evolving cyber threats. Several promising avenues for future research emerge from this study. Integration with emerging technologies such as blockchain and federated learning can be explored to enhance the security and privacy aspects of the framework. Investigating the incorporation of explainable AI techniques to improve the interpretability of intrusion detection models is another potential direction. Future work can focus on studying the scalability and performance of the proposed framework in large-scale, heterogeneous IoT environments, optimizing resource allocation, load balancing, and distributed processing techniques to ensure efficiency and effectiveness in handling massive volumes of IoT data. Adapting the framework to handle evolving threat landscapes, including zero-day attacks and emerging attack vectors, is a crucial area for future investigation. Developing unsupervised anomaly detection techniques, transfer learning approaches, and continuous model updates based on real-time threat intelligence can enhance the framework’s resilience. Moreover, integrating the intrusion detection framework with automated response mechanisms can enable proactive defence against cyber threats, leveraging machine learning and AI techniques to initiate appropriate countermeasures based on detected threats autonomously. Fostering collaboration between academia and industry is essential for translating research findings into practical solutions. Developing partnerships and joint initiatives to pilot the proposed framework in real-world fog computing and IoT deployments can provide valuable feedback and insights for further refinement and enhancement. By exploring these future research directions, we aim to contribute to the development of more secure, resilient, and intelligent intrusion detection solutions for fog computing and IoT networks. As the adoption of these technologies continues to accelerate, the importance of robust intrusion detection frameworks will only increase, making this an exciting and impactful area for ongoing research and innovation.
